# Heat Priming Induces *Trans*-generational Tolerance to High Temperature Stress in Wheat

**DOI:** 10.3389/fpls.2016.00501

**Published:** 2016-04-14

**Authors:** Xiao Wang, Caiyun Xin, Jian Cai, Qin Zhou, Tingbo Dai, Weixing Cao, Dong Jiang

**Affiliations:** ^1^National Technology Innovation Center for Regional Wheat Production/National Engineering and Technology Center for Information Agriculture/Key Laboratory of Crop Physiology and Ecology in Southern China, Ministry of Agriculture, Nanjing Agricultural UniversityNanjing, China; ^2^Rice Research Institute, Shandong Academy of Agricultural SciencesJinan, China

**Keywords:** wheat, heat treatment, photosynthesis, antioxidant activity, physiological analysis

## Abstract

Wheat plants are very sensitive to high temperature stress during grain filling. Effects of heat priming applied to the first generation on tolerance of the successive generation to post-anthesis high temperature stress were investigated. Compared with the progeny of non-heat primed plants (NH), the progeny of heat-primed plants (PH) possessed higher grain yield, leaf photosynthesis and activities of antioxidant enzymes and lower cell membrane damage under high temperature stress. In the transcriptome profile, 1430 probes showed obvious difference in expression between PH and NH. These genes were related to signal transduction, transcription, energy, defense, and protein destination and storage, respectively. The gene encoding the lysine-specific histone demethylase 1 (*LSD1*) which was involved in histone demethylation related to epigenetic modification was up-regulated in the PH compared with NH. The proteome analysis indicated that the proteins involved in photosynthesis, energy production and protein destination and storage were up-regulated in the PH compared with NH. In short, thermos-tolerance was induced through heritable epigenetic alternation and signaling transduction, both processes further triggered prompt modifications of defense related responses in anti-oxidation, transcription, energy production, and protein destination and storage in the progeny of the primed plants under high temperature stress. It was concluded that *trans*-generation thermo-tolerance was induced by heat priming in the first generation, and this might be an effective measure to cope with severe high-temperature stresses during key growth stages in wheat production.

## Introduction

Wheat is a worldwide staple food crop. However, wheat yield fluctuates largely due to abiotic stress. Along with the global climate change, the occurrences of extreme weather events such as heat and drought stresses have significantly increased, in terms of frequency, extent and duration (Field et al., 2014). In wheat, the optimum growth temperature is around 21°C during reproductive growth (Porter and Gawith, 1999). Temperatures higher than 33°C in this stage lead to significant decline of leaf photosynthesis and imbalance of reductive-oxidative state, reduced grain filling duration, and obvious yield loss (Ugarte et al., 2007; Barlow et al., 2015).

Pre-exposure of plants to mild stress may bring about “stress memory” that facilitates a fast protective response to a subsequent stress event (Bruce et al., 2007; Boyko and Kovalchuk, 2011). Stress memory has been defined as genetic, epigenetic and physiological changes under stress conditions, and it modifies responses to reoccurring stress in the same generation (in-generation) or in the next generation (*trans*-generation) (Boyko and Kovalchuk, 2011).

In-generation stress memory has been found to be associated with enhanced tolerance to various biotic or abiotic stresses in many plant species (Conrath et al., 2006). Molecular analysis shows that stress memory induced by pathogen attack (Kovalchuk et al., 2003; Conrath, 2011), are associated with epigenetic variations (such as DNA methylation, chromatin remodeling, or small interfering RNAs), signaling proteins accumulation, chromatin modifications, and primary metabolism alterations (Bruce et al., 2007; Conrath, 2011). Our previous study found that moderate heat priming applied before anthesis effectively alleviated the impacts of severe high temperature stress occurring during grain filling. This alleviation was attributed to a lessened damage by oxidative stress at both leaf and sub-cellular levels, and thus a large maintainence of photosynthesis (Wang et al., 2011, 2014a), and to an enhanced carbohydrate remobilization from stems to grains (Wang et al., 2012).

It is very interesting that priming inducing stress tolerance has been shown to persist to the descendants hereby improving tolerance to stress reoccurring in the next generation (Molinier et al., 2006; Ton et al., 2007; Rasmann et al., 2012). This improved stress tolerance is attributed to enhanced expression of salicylic acid mediated defense-related genes (Slaughter et al., 2012), and epigenetic modifications (Pieterse, 2012) in descendants of the primed plants. Besides, it has been proven that *trans*-generational priming, triggered by bacterial infections, is transmitted by hypomethylation of salicylic acid – dependent genes to the next generation (Luna et al., 2012). In addition, earlier study reported that the progeny of primed *Arabidopsis* plants are more tolerant to several abiotic stresses, and that the tolerance is related to substantially modified gene expressions, increased homologous recombination frequency, and increased DNA methylation (Boyko et al., 2010). However, the underlying mechanisms of *trans*-generational stress tolerance induced by priming remain largely unknown (Walter et al., 2011).

In the present study, it was hypothesized that heat primed wheat plants could transfer the high temperature stress memory to their progeny, via modifying expressions of a set of genes and proteins. Namely, the differential expression of genome and proteome would be found in the progeny of the primed plants during high temperature stress and contributed their tolerance to high-temperature stress during grain filling. The effects of *trans*-generation priming were analyzed at physiological, transcriptomic and proteomic levels and the underlying mechanisms of these effects were discussed.

## Materials and Methods

### Plant Growth Conditions and Treatments

Pot experiments (25 cm in diameter and 22 cm in depth) were conducted at the Experimental Station of Nanjing Agricultural University, Nanjing, China (32°30′N and 118°42′E). Seeds of a commercial winter wheat (*Triticum aestivum* L. cv. Yangmai 16) were used in this study. During the first generation, wheat plants were divided into two groups: one group was not primed (N) while the other was heat-primed (P) at pre-anthesis stages (at a day/night temperature of 32/28°C for 2 days at the seven-leaf stage and the nine-leaf stage, respectively) and post-anthesis stage (34/30°C for 7 days started at the 10th day after anthesis, DAA). At maturity, seeds from both groups were harvested.

Plants (the progeny) grown from seeds harvested in each group were further divided in two sub-groups from 10 DAA: one was subjected to high temperature stress at a day/night temperature of 34/30°C, while the other was set at 26/22°C for 6 days. During both priming and high-temperature stress, plants under different treatments were moved into separate growth chambers. The photosynthetically active radiation (PAR) was set at 400 μmol m^-2^ s^-1^ with a day/night cycle of 14/10 h. At the end of the priming and high-temperature stress events, plants were moved out of the chambers.

In total four treatments were established: NC, progeny of non-primed plants (N) without post-anthesis high temperature stress; NH, progeny of non-primed plants with post-anthesis high temperature stress; PC, progeny of primed plants (P) without post-anthesis high temperature stress; and PH, progeny of primed plants with post-anthesis high temperature stress. The experiment was arranged in a completely randomized block design with three biological replicates.

### Grain Yield

At maturity, plants were harvested and grains were collected. Grain yield per pot and 1000-grain weight were determined. Three pots for each treatment were harvested as three biological replicates.

### Photosynthesis

On the last day of the high temperature stress, A/C_i_ curve (net carbon assimilation rate *versus* intercellular CO_2_ concentration) of the flag leaf was measured using a LI-6400 system (LI-COR Biosciences, Lincoln, NE, USA) embedded with the “A/C_i_ curve” auto-program and equipped with light-emitting diode (LED) source at a light level of 1200 μmol m^-2^ s^-1^. Three replicates (leaves) were taken for each treatment. Non-linear regression (Sharkey et al., 2007) was used to estimate the maximum carboxylation velocity of Rubisco (V_max_), the maximum electron transport rate (J_max_), and the saturated net photosynthesis rate (A_sat_).

### Pigments Concentration

The pigments measurements were performed following the method of Wang et al. (2011). Briefly, at the end of high temperature stress, 0.1 g fresh flag leaves were sliced and incubated in 50 ml of acetone and anhydrous extraction solution (1:1). The pigments were extracted at 25°C in dark for 24 h. Chlorophyll and carotenoid concentrations were measured spectrophotometrically (UV-2401, Shimadzu Crop., Japan) at 663, 645, and 470 nm, respectively.

### Antioxidant Enzyme Activities and Malondiaidehyde (MDA) Content

Superoxide dismutase and POD were extracted according to Tan et al. (2008). In brief, at the end of high-temperature stress, 0.5 g fresh flag leaves was sliced and homogenized in 5 ml of extraction buffer (50 mM potassium phosphate, pH 7.0, including 0.4% PVP) at 4°C. The extract was then centrifugated at 10, 000 *g* for 15 min at 4°C. The supernatant was collected for the measurement of enzyme activities and MDA content.

The activities of SOD and POD were assayed according to Tan et al. (2008). In brief, at the end of high-temperature stress, 0.5 g fresh flag leaves was sliced and homogenized in 5 ml of extraction buffer (50 mM potassium phosphate, pH 7.0, including 0.4% PVP) at 4°C. The extract was then centrifugated at 10, 000 *g* for 15 min at 4°C. The supernatant was collected for the measurement of enzyme activities and MDA content. The activities of SOD and POD were assayed according to Dhindsa et al. (1981).

### Transcriptome Analysis

At the end of high temperature stress, fresh flag leaves were used for gene transcriptome analysis with the Affymetrix GeneChips (Affymetrix, Santa Clara, CA, USA). The analysis was conducted in the Capitalbio Corporation in Beijing according to the instructions for Affymetrix (Affymetrix GeneChip Expression Analysis Technical Manual, Affymetrix). GeneChip Operating Software 1.4 was used to analyze the hybridization data under the criteria of at least 2.0-fold changes (log_2_ values), with a false discovery rate <0.05 at gene expression level. Functional annotation was performed based on *Arabidopsis thaliana* genome databases.

### Proteome Analysis

Protein was extracted using a modified version of the trichloroacetic acid acetone precipitation method as described by Rinalducci et al. (2011). Briefly, at the end of high temperature stress, fresh flag leaves were taken and finely ground in liquid nitrogen, precipitated overnight by addition of 10 volumes of cold acetone containing 10% (w/v) trichloroacetic acid acetone, 1 mM PMSF, and 10 mM DTT at -40°C. The extraction was centrifugated at 20, 000 *g* for 20 min at 4°C. The pellet was washed three times with cold acetone containing 1 mM PMSF and 10 mM DTT and incubated at -40°C for 1 h. The purified protein pellets were dissolved in lysis buffer containing 7 M urea, 2 M thiourea and 4% (w/v) CHAPS, and were then centrifugated at 30,000 *g* for 30 min at 4°C. The supernatant was collected and protein concentration was determined by the Bradford method (Branford, 1976). Rehydration, focusing, and strip equilibration were performed according to the procedure developed by Wang et al. (2014b). The two-dimensional gels were stained by silver nitrate according to the method of Lin et al. (2008).

PDQuest software (version 8.0, Bio-Rad) was used for the two-dimensional gel images. Those differentially expressed protein spots with at least 1.5-fold change over the control were used for further analysis. Protein identification was carried out by the MALDI-TOF/TOF mass spectrometer (ABI 4800). The MASCOT database search engine^1^ was used to search for peptide mass lists from the obtained spectra against the NCBInr database^2^ according to the procedure of Wang et al. (2014b).

### Statistical Analysis

Data were subject to the one-way ANOVA with the Sigmaplot 11.0 (Systat Software). Significantly different means of the measured data were separated at *P* < 0.05 by the Duncan’s multiple range test.

## Results

### Progeny of Heat-Primed Plants Was More Tolerant to Post-anthesis High-Temperature Stress

Post-anthesis high-temperature stress caused significant reduction in wheat grain yield and grain weight. However, progeny of primed plants (PH) showed much less losses in grain yield and grain weight than that of the non-primed plants (NH), compared with control NC (**Table 1**). Similarly, the contents of chlorophyll and carotenoid were much higher in the PH than in the NH plants. In addition, both photosynthetic rate and leaf dry matter remobilization were more active in the PH than in the NH plants. There was no significant difference in V_max_ between PH and NH, while J_max_ and A_sat_ decreased much more in the NH than in the PH plants. In addition, grain yield, plant dry matter remobilization rate, and photosynthesis related parameters showed no significant differences between the PC and the NC plants.

**Table 1:** Progeny of heat primed plants showed better physiological performance than that of non-primed plants under high-temperature stress during grain filling.

Measurements	Treatments			Fold change	
	NC	NH	PH	PC	PH/NH
**Photosynthesis related parameters**					
Chla (mg g^-1^ fresh weight)	4.1^a^	2.4^b^	3.7^a^	4.0^a^	1.5
Car (mg g^-1^ fresh weight)	0.72^a^	0.53^b^	0.61^ab^	0.69^a^	1.2
V_max_ (μmol m^-2^ s^-1^)	99.0^a^	85.5^b^	89.0^ab^	97.1^a^	1.0
J_max_ (μmol m^-2^ s^-1^)	123.1^a^	97.5^b^	128.5^a^	125.4^a^	1.3
A_sat_ (μmol CO_2_ m^-2^ s^-1^)	32^a^	26^b^	34^a^	32^a^	1.3
**Remobilization amount of pre-anthesis stored dry matter (RAP)**					
Leaf RAP (mg stem^-1^)	247.7^a^	219.3^b^	243.2^a^	241.0^ab^	1.1
Stem RAP (mg stem^-1^)	229.3^a^	198.7^b^	210.0^b^	231.1^a^	1.1
Hull and rachis RAP (mg stem^-1^)	56.3^a^	41.0^b^	46.7^b^	54.7^a^	1.1
**Grain yield**					
Kernels per spike	34.4^a^	31.5^a^	31.3^a^	32.9^a^	1.0
Thousand grain weight (g)	33.7^a^	28.3^b^	32.9^a^	33.7^a^	1.2
Yield (g/pot)	8.1^a^	5.7^b^	7.2^a^	7.4^a^	1.3

*Chla, chlorophyll concentration; Car, carotenoid concentration; RAP, V_max_, maximum carboxylation velocity of Rubisco; J_max_, maximum photosynthetic electron transport rate, A_sat_, saturated net photosynthesis.*

*Different small letters within a row mean significant difference (P < 0.05).*

**FIGURE 1:**
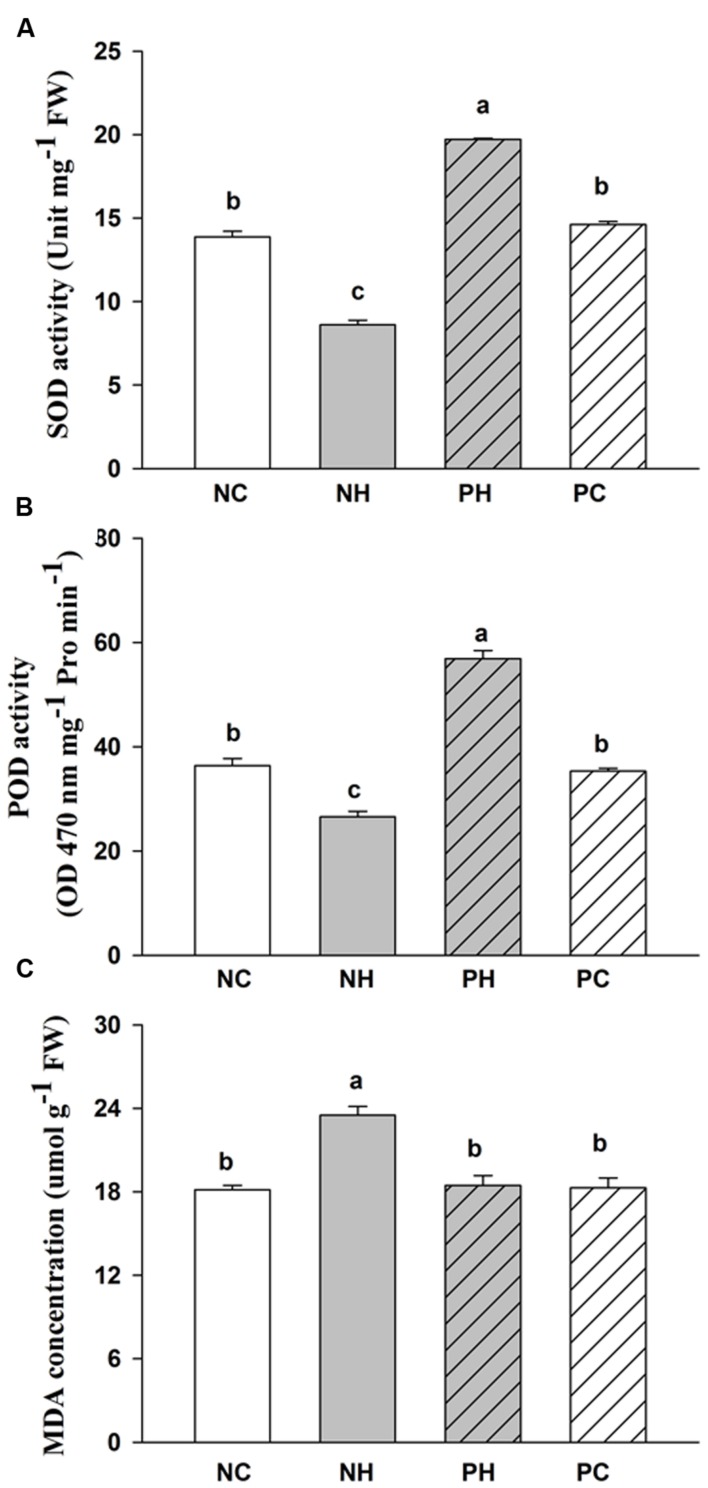
**Progeny of heat primed plants are more tolerant to high-temperature stress applied during grain filling than that of non-primed plants as exemplified by up-regulated activities of antioxidant enzymes. (A)** SOD; **(B)** POD; **(C)** MDA; NC, progeny of non-primed plants without post-anthesis high temperature stress; NH, progeny of non-primed plants with post-anthesis high temperature stress; PC, progeny of primed plants without post-anthesis high temperature stress; and PH, progeny of primed plants with post-anthesis high temperature stress.

The activities of SOD and POD respectively decreased by 30.1 and 27.0% in the NH, but increased by 24.1 and 56.4% in the PH, as compared with the NC plants (**Figure 1**). The content of MDA increased by 29.5% in the NH, but showed no significant change in the PH, as compared to the NC plants. The data implied a lower cell membrane lipid peroxide level in the progeny of the primed plants, which was the result of higher capacity of scavenging reactive oxygen species in the progeny of the primed plants than in progeny of the non-primed plants under post-anthesis high-temperature stress.

### *Trans*-generational Priming Effect Was Related to Transcriptome Modifications

The hierarchical clustering of gene transcription in wheat leaves under post-anthesis high-temperature stress is shown in Supplementary Figure S1. According to the similarities in gene expression patterns, the differentially expressed genes were classified into two groups as the non-high-temperature stress group (NC vs. PC) and the high-temperature stress group (NH vs. PH). In relation to the NC plants, fewer genes were differentially (up- and down-) regulated in the PH than in the NH plants, and more genes were down-regulated in the NH than in the PH plants, as compared with the NC plants (Supplementary Figure S2). This indicated that fewer genes were manipulated in the progeny of the primed plants than in that of the non-primed plants in responding to post-anthesis high-temperature stress. A total of 1430 probes were significantly differentially expressed between PH and NH plants, with 906 up-regulated and 524 down-regulated (Supplementary Table S1). Furthermore, 157 probes were up-regulated while 108 probes were down-regulated in the PC compared to the NC plants (Supplementary Table S2).

**FIGURE 2:**
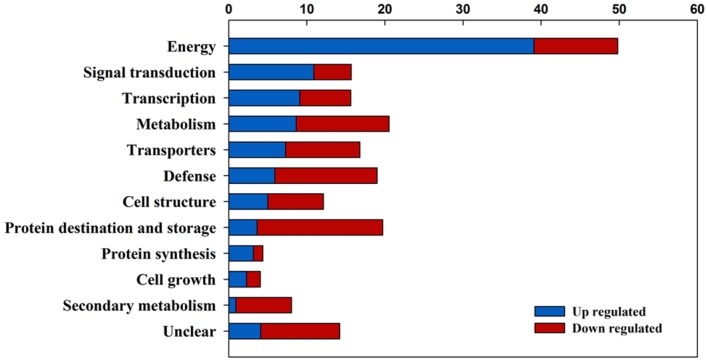
**The summarized gene ontology analysis of the differently expressed genes between progeny of primed plants (PH) and non-primed plants (NH) under heat stress during grain filling.** The blue bars stand for the up-regulation of expressions of clustered genes in PH relative to NH; the red bars stand for the down-regulation of expressions of clustered genes in PH relative to NH.

The differentially expressed genes between the PH and the NH plants were classified into groups related to energy, epigenetic, signal transduction, defense, metabolism, transporters, transcription, protein destination and storage, cell structure, protein synthesis, cell growth, secondary metabolism based on their biological functions (Bevan et al., 1998), as shown in Supplementary Table S1. The description of the biological function of the differentially expressed genes between the PC and the NC plants is shown in Supplementary Table S2. Moreover, the processes of signal transduction, transcription, energy, defense, and protein destination and storage showed significant difference between the PH and the CH plants (**Figure 2**).

### Signal Transduction Related Genes

Several classes of genes encoding proteins involved in abiotic stress signaling pathways were up-regulated in the PH as compared with the NH plants. These included the genes encoding Ca^2+^ signaling pathway (Ca^2+^-binding protein 1, calcium sensing receptor, calcium-binding EF hand family protein, and so on), the mitogen-activated protein kinase cascade (MAP kinase 20), and other protein kinases (protein kinase superfamily protein, membrane protein, and concanavalin A-like lectin protein kinase family protein) (Supplementary Table S1).

The genes encoding hormone-mediated signaling pathways were differently regulated between the PH and the CH plants. Here, the genes encoding ABA mediated signaling pathway (casein kinase 1-like protein and alpha dioxygenase) was differently regulated between the PH and the NH plants. The genes encoding IAA mediated signaling pathway (N-MYC down-regulated-like 1) and the cytokinins mediated signaling pathway (CTK, histidine kinase 3 and Ribosomal protein L6 family) were up-regulated in the PH as compared with the CH plants. The genes encoding ethylene signaling pathway (multiprotein bridging factor 1C) was down-regulated in the PH than in the CH plants. The genes encoding JA biosynthesis pathway (Lipoxygenase 2 and allene oxide cyclase 1, 4) was differently expressed between the PH and the CH plants. At the same time, jasmonate-zim-domain protein 1, a suppressor of JA signaling, was down-regulated in PH, compared with CH. Some other protein kinases (lectin protein kinase family protein, receptor like protein kinase 32, and Leucine-rich receptor-like protein kinase family protein) encoding hormone-mediated signaling pathways were up-regulated in the PH as compared with the NH plants (Supplementary Table S1).

### Transcription and Energy Related Genes

The second largest up-regulated group in the PH as compared with the NH plants was the transcription related genes. These included genes involved in RNA binding (ribonuclease T2 family protein, chloroplast stem-loop binding protein, and so on), RNA processing (ribonucleotide reductase 1, U2 small nuclear ribonucleoprotein A, NOP56-like pre-RNA processing ribonucleo protein, D111/G-patch domain-containing protein), splicing [RNA-binding (RRM/RBD/RNP motifs) family protein] and methylation (Arginyl-tRNA synthetase, putative mitochondrial RNA helicase, and so on). Importantly, the genes encoding lysine-specific histone demethylase involved in the chromatin modification and epigenesis were significantly up-regulated in the PH as compared with the NH plants (Supplementary Table S1).

Most genes classified into the energy group were up-regulated in the PH as compared with the NH plants (Supplementary Table S1), e.g., genes encoding the photosynthetic enzymes (PsbQ-like 2, the light harvesting complex of photosystem II,photosystem I light harvesting complex, and so on), glycolysis (aldolase superfamily protein, starch synthase, beta glucosidase, and so on), and ATP production (ATP synthase alpha/beta family protein, ATPase gamma subunit protein, and DEAD/DEAH box helicase).

### Redox and Stress Response Related Genes

The anti-oxidation related genes encoding the pyridine nucleo tide-disulphide oxidoreductase family protein, aldehyde dehyd rogenase, ascorbate POD etc., were up-regulated in the PH as compared with the NH plants. The genes encoding the alternative oxidase, POD superfamily protein, glutathione *S*-transferase family protein etc., were down-regulated in the PH as compared with the NH plants (Supplementary Table S1).

In addition, some abiotic stress responding genes, such as those encoding the drought-responsive family protein, the putative GTP diphosphokinase RSH1 were up-regulated in the PH as compared with the NH plants. The genes in response to cold stress (germin-like protein 4), biotic stress (ribonuclease 1, pathogenesis related protein, and adenine nucleotide alpha hydrolases-like superfamily protein, bifunctional nuclease I), and drought stress (LEA) were down-regulated in the PH as compared with the NH plants (Supplementary Table S1).

### Protein Destination and Storage Related Genes

The genes encoding proteinases (cysteine proteinases superfamily protein, amino acid permease 1, and so on) and chaperones (PDI-like 1-4, TCP-1/cpn60 chaperonin family protein, and cyclophilin 38) were up-regulated in the PH as compared with the NH plants (Supplementary Table S1). Some genes encoding the cysteine proteinases superfamily protein and eukaryotic aspartyl protease family protein were down-regulated in the PH as compared with the NH plants. The genes encoding protease inhibitors (serine protease inhibitor, and potato inhibitor I-type family protein), chaperones (chaperone DNAJ-domain superfamily protein, calcium-binding EF-hand family protein, and so on), and heat shock proteins (HSP21, HSP101, HSP70, and so on) were also down-regulated in the PH as compared with the NH plants (Supplementary Table S1).

### *Trans*-generational Priming Effect Was Related to Proteome Modifications

It was found that 15 spots were differentially regulated in the PH and the NH plants under post-anthesis high-temperature stress, of which nine were successfully identified (Supplementary Figure 3). The identified proteins were classified into groups associated with energy, protein destination and storage, metabolism and cell structure (**Table 2**). Of the nine identified spots, seven were up-regulated in the PH as compared with the NH plants, including spot 1204 (phosphoribulokinase), spot 2403 (ribulose 1,5-bisphosphate carboxylase activase), spots 1703 and 1704 [protein disulphide isomerase (PDI)], spot 3503 (ATP synthase sub-unit beta), spot 3807 (vacuolar proton-ATPase subunit A), and spot 6803 (methionine synthase); the other two were down-regulated, including spot 3806 (HSP70), and spot 4503 (xylose isomerase). Only one spot (1703, PDI) was observed to be down-regulated in the PC as compared with the NC plants.

**Table 2:** Differentially expressed proteins between progenies of primed and non-primed plants under high-temperature stress during grain filling.

Spot	PC/NC	PH/NH	Protein name	Taxonomy	GI No.	Theory data (Mr/pI)	Experimental date (Mr/pI)	Score	SC (%)	Classification
1204	0.95	2.04	Phosphoribulokinase	*Triticum aestivum*	21839	45.4/5.8	40.5/5.0	194	8	Energy
2403	0.94	2.02	Rubisco activase	*Hordeum vulgare*	167096	47.3/8.6	41.2/5.3	489	18	Energy
3503	1.01	1.51	ATP synthase subunit beta	*Glycine max*	356536246	59.9/5.8	55.8/5.5	434	13	Energy
3807	0.98	1.51	Vacuolar proton-ATPase subunit A	*Triticum aestivum*	90025017	68.8/5.2	73.6/5.5	515	14	Energy
1703	0.65	2.3	Protein disulfide isomerase 3 precursor	*Triticum aestivum*	13925728	56.9/5.0	67.5/5.1	225	10	Protein destination and storage
1704	0.79	2.39	Protein disulfide-isomerase	*Triticum aestivum*	1709620	56.7/5.0	64.5/5.1	83	5	Protein destination and storage
6803	1.01	1.51	Methionine synthase	*Hordeum vulgare*	50897038	84.8/5.7	86.7/6.3	204	5	Metabolism
3806	1.01	0.49	HSP70	*Triticum aestivum*	2827002	71.4/5.4	74.8/5.4	586	14	Protein destination and storage
4503	1.12	0.47	Xylose isomerase	*Hordeum vulgare*	1296807	53.9/5.3	55.0/5.7	196	9	Cell Structure

## Discussion

It is well accepted that pathogen-induced stress memory can be transferred to descendants in plants (Conrath et al., 2002, 2006; Niinemets, 2010). The epigenetic mechanisms are believed to be involved in the inheritance of tolerance to biotic stress in *trans*-generational priming (Balmer et al., 2015). To our best knowledge, very few studies have been reported on the *trans*-generational epigenetic inheritance induced by abiotic stress (Hauser et al., 2011). Thereafter, we primed the wheat plants during the first generation, and then subjected the progeny plants to high-temperature stress during grain filling. The *trans*-generational alleviation effects of heat priming on high-temperature stress occurring in the next generation were evaluated.

Under high-temperature stress during grain filling, the progeny of the primed plants (PH) showed significantly higher grain yield, which was mainly attributed to the higher grain weight of the primed plants than that of the non-primed plants (NH). The greater grain weight could be resulted from the higher leaf photosynthetic rate and dry matter translocation in the PH than in the NH plants. These results suggested the improved tolerance to high-temperature stress in the progeny of the primed plants than in the progeny of the non-primed plants. Alternatively, the priming effect during the first generation was inherited by their progeny which could enhance their thermos-tolerance.

### Epigenetic Modifications

There are growing evidence suggesting that epigenetic processes, including heritable DNA methylation, histone modification or chromatin re-modeling independent of DNA sequence changes (Kouzarides, 2007; Mathieu et al., 2007), are closely related to the transfer of the stress memory to the progeny (Molinier et al., 2006; Bruce et al., 2007; Chinnusamy and Zhu, 2009; Boyko and Kovalchuk, 2011; Iwasaki and Paszkowski, 2014). Lysine-specific histone demethylase 1 (LSD1) is a type of histone demethylases that specifically demethylates histone H3 lysine 4 (H3K4) me1/2 (Shi et al., 2004) and is associated with transcriptional repression and activation in stress response (Yuan et al., 2013). At the same time, changes in nucleic acid gene expression also affect DNA methylation status (Yaish et al., 2009). In this study, the genes encoding LSD1, putative nucleic acid methyl transferases and binding proteins, RNA methyltransferase, and ribosomal RNA FtsJ-like methyltransferase were expressed much higher levels in the progeny of the primed plants than in those of the non-primed plants under post-anthesis high-temperature stress (**Table 2**). This suggested that the histone demethylation and the global methylation of DNA were involved in the *trans*-generational memory. Namely, these epigenetic marks enabled the progeny of the primed plants to induce stress related genes to better conquer the high-temperature stress than progeny of the non-primed plants.

### Signaling Transduction

The perception and transmission of stress signals are the first and the most important aspects for plant in responses to environmental perturbations. Calcium signaling, receptor-like protein kinases (RLKs) and other protein kinases are believed to play fundamental roles in stress signal transduction processes in plants (Harper et al., 2004). The increased content of cytosolic Ca^2+^ under higher temperature could be linked to the acquisition of thermo-tolerance by transducing high temperature-induced signals to MAPK (Gong et al., 1997; Larkindale and Knight, 2002; Padmalatha et al., 2012). The up-regulation of genes involved in Ca^2+^ and MAPK signaling pathways under water deficit suggests the induction of the signaling pathways, which in turn activates stress responsive target genes to overcome drought stress (Zhu, 2002). In agreement with this, here the genes involved in the Ca^2+^ and MAPK signaling pathways (MAPK 20) were more highly expressed in the PH than in the NH plants, suggesting that the PH plants could better activate the Ca^2+^ signaling pathways to induce expression of the stress response genes than did the NH plants.

Plant hormones play significant roles in signaling pathway to regulate abiotic stress response in plants, independently, synergistically or antagonistically (Bari and Jones, 2009; Park et al., 2014). ABA induction is an important component of thermo-tolerance response involved in biochemical pathways (Maestri et al., 2002). Casein kinase 1-like (CKL) positively mediate ABA signaling (Cui et al., 2014), but alpha dioxygenase (*APD*) negatively regulates ABA-mediated signaling pathways. In addition, the circadian clock associated 1 (CCA) (a key phytochrome regulation factor) and bZIPTFs (acting as major transcription factors) regulates the expressions of ABA- dependent genes under abiotic stresses (Fujita et al., 2013). Here, the up-regulated *CKL2, CCA1, bZIPTFs* and down-regulated *APD* in the PH compared with the CH plant suggested that the ABA-signaling pathway was induced in progeny of the primed plants.

Histidine kinases (HKs) and ribosomal protein L6 family (RPL) mediate responses to the cytokinin activated signaling pathway (Inoue et al., 2001), and have a positive impact on stress tolerance by influencing many downstream signaling factors (Osakabe et al., 2013). Here, the genes encoding HKs and ribosomal protein L6 family were much highly up-regulated in the PH and in the PC plants as compared with the NH and the NC plants. This suggested that the induced cytokinin signaling pathway in the PH plants might be inherited from their primed parents. The N-MYC down-regulated-like 1, the positive regulators of auxin transport in a G protein mediated pathway (Mudgil et al., 2009), was also up-regulated in the PH as compared with the NH plants.

The above results indicated that the enhanced heat tolerance in the progeny of the primed plants might be related to the induction of expression of genes involved in ABA, cytokinin and auxin signaling transduction pathways.

In addition, JA is reported to be closely related to the heritable tolerance induced by biotic stress (Luna et al., 2012). In this study, lipoxygenase 2 (LOX) and allene oxide cyclase 1 (AOC) involved in the JA biosynthesis, were differently regulated between the PH and the CH plants. However, jasmonate-zim-domain protein 1, the suppressor of JA signaling transduction (Thines et al., 2007), was down-regulated in the PH as compared with the NH plants. This indicated that JA signaling transduction was enhanced in the progeny of the primed plants under post-anthesis high temperature stress.

Multi-protein bridging factor 1C (MBF) involved in the ethylene signaling transduction was down-regulated in the PH as compared with the NH plants. Reversely, genes encoding ethylene-forming enzymes were up-regulated in the PC as compared with the NC. It seemed that the ethylene signaling did not need to be re-triggered in the progenies grown under post-anthesis high-temperature stress, implying the ethylene signaling pathway induced in the parent plants by heat-priming could have been transferred to their progenies.

In literature, the RLK family is commonly recognized as innate immune receptors in plants and is also involved in cell-to-cell signaling processes (Park et al., 2014). The largest subgroup of this family is the leucine rich repeat RLK (LRR-RLKs) family (Gust and Felix, 2014; Halter et al., 2014). Phytosulphokin receptor 1 is a member of the LRR-RLK family (Kwezi et al., 2011). These RLKs with wall-bound extracellular domains may function in sensing the integrity or dynamic status of cell wall (Shiu and Bleecker, 2001). In the present study, genes encoding RLKs and other kinase related proteins (protein kinase, concanavalin A-like lectin protein kinase family protein and membrane protein) were up-regulated in the PH as compared with the NH plants, indicating that up-regulated protein kinase related proteins could confer the progeny of the primed plants more robust response to high-temperature stress than the progeny of the non-primed plants.

### Photosynthesis and Detoxification

As the most important factor determining crop yield, photosynthesis is also the most sensitive physiological process to high temperature stress (Salvucci and Crafts-Brandner, 2004). Here, the photosynthesis related genes were significantly up-regulated in the PH plants, consistent with the higher photosynthetic rate in the PH as compared with the NH plants. It has been reported that the disturbed photosynthetic CO_2_ fixation due to heat stress is resulted from the down-regulation of proteins related to phosphoribulokinase and rubisco activase, the key regulatory enzymes in the Calvin Cycle (Lee et al., 2007). Therefore, higher expressions of phosphoribulokinase and rubisco activase could indicate greater capacities of photosynthesis maintenance and acclimation under heat stress (Zou et al., 2011). In this study, the protein abundance of phosphoribulokinase and rubisco activase was up-regulated in the PH as compared with the NH plants (**Table 2**). The up-regulated expressions of photosynthesis related genes and proteins were paralleling with the maintenance of photosynthetic capacity in the PH plants (**Table 1**), suggesting that the protection of photosynthetic process contributed to the *trans*-generation thermo-tolerance in the progeny of the primed plants.

The high level of ATP synthase subunit beta and vacuolar proton-ATPase subunit A are reported to be closely correlated with the enhanced energy under acclimation to salt stress (Guo et al., 2012). In this study, the ATP synthase subunit beta and vacuolar proton-ATPase subunit A were up-regulated in the PH but down-regulated in the NH plants as compared with the NC plants. This implied that the enhanced energy supply could be induced in the progeny of the primed plants in response to post-anthesis high-temperature stress.

Cellular redox homeostasis has been shown to be crucial in maintaining the normal physiological activity of plants (Sen and Packer, 1996). In the present study, the activities of SOD and POD were much higher in the PH than in the NH plants, leading to a lower cell membrane peroxidation rate in the PH than in the NH plants (**Table 1**). Accordingly, genes related to oxidation/reduction processes, such as genes encoding APX, GPX and thioredoxin were up-regulated in the PH as compared with the NH plants. APX and GPX can directly detoxify H_2_O_2_ to H_2_O (Mittler, 2002). Our previous studies have demonstrated that APX could serve as the central candidate for drought priming induced tolerance in the same generation in wheat (Wang et al., 2014b). Thioredoxin superfamily protein also plays a crucial role in biological responses against oxidative stress (Tanaka et al., 2000). Here, other enzymes related to oxidation/reduction processes (pyridine nucleotide – disulphide oxidoreductase family protein, and FAD/NAD(P) – binding oxidoreductase family protein) were up-regulated in the PH plants, which was probably related to the requirement for maintaining a better balance between NAD(P) and NAD(P)H for ATP synthesis in the PH as compared with the NH plants.

The POD superfamily are reported to be involved in many functions such as generation and regulation of ROS and oxidation of various substrates, and these PODs are significantly induced by abiotic stress or biotic stress in plants (Passardi et al., 2005). Lactoylglutathione lyase/glyoxalase I family protein is involved in the glutathione-based detoxification of methylglyoxal, a toxic by-product of carbohydrate and amino acid metabolism (Witzel et al., 2009). In this study, the genes encoding POD superfamily protein, glutathione *S*-transferase family, and lactoylglutathione lyase/glyoxalase I family protein were up-regulated in the NH as compared with the PH plants. In addition, the genes encoding POD superfamily and cytochrome P450, which are involved in the plant defense, were up-regulated in the PC as compared with the NC plants.

Furthermore, aldehyde dehydrogenase, an efficient scavenger of reactive oxygen species and an inhibiting enzyme of lipid peroxidation (Kotchoni et al., 2006), and serine transhydroxymethyltransferase, that is involved in controlling cell damage caused by abiotic stress (Oliveira et al., 2015), both were up-regulated in the PH as compared with the NH plants. Bifunctional nuclease I, which is involved in programmed cell death and senescence (Pérez-Amador et al., 2000), was down-regulated in the PH as compared with the NH plants. These results were consistent with the higher activities of the antioxidant enzymes and lower content of MDA in the PH than in the NH plants. This indicated that the progeny of the primed plants could facilitate positive defense in responses to high-temperature stress through regulation of expression of redox related genes, resulting in less cell membrane damage. It could be inferred from these results that the cellular defense responses might be activated not only via high-temperature stress but also via *trans*-generational transfer of the priming memory and might be expressed in an accelerated manner after perception and transduction of the high-temperature stress signals, all of which might be a process affected by epigenetic modifications.

### Protein Destination and Storage Related Genes

The PDI family, which is traditionally regarded as endoplasmic reticulum (ER) enzyme involved in protein folding, serves as a molecular chaperone and also catalyzes reactions of oxidation and reduction (Porter et al., 2015). In this study, the expression of PDI encoding genes and protein abundance were reduced in the NH plants, but were not significantly affected in the PH plants as compared with the NC controls. The greater maintenance of gene expression and protein abundance of PDI in the PH than in the NH plants indicated that PDI could play important roles in assisting protein folding and in balancing oxidation and reduction in the progeny of primed plants.

Heat shock proteins are crucial in the thermo-protection of plant cells under detrimental heat stress (Iba, 2002). Many studies show that HSPs are molecular chaperones to ensure native configuration and functionality of cell proteins under heat stress. Considerable evidences show that thermo-tolerance acquisition is directly related to the synthesis and accumulation of HSPs (Bruce et al., 2007). HSPs are known to be quite normal proteins in response to heat stress at transcription and protein level in both heat sensitive and tolerant varieties (Wang et al., 2015). In this study, expressions of HSPs encoding genes and abundance of HSP70 protein were up-regulated in both the PH and NH plants as compared with the NC controls. However, the NH plants showed significantly higher expression than did the PH plants. This suggested that the up-regulation of HSP was a common response to heat stress, and that the over-accumulations of HSPs could be related to the much more severe cell damage in the NH than in the PH plants. It might also indicate that the over-expression of HSPs could not be involved in the *trans*-generational stress memory transfer.

The weak correlation between mRNA expression and protein abundance is found in many studies (Amiour et al., 2012; Fernie and Stitt, 2012). This weak correlation is probably the result of regulatory processes of gene expression, various post-translation modifications and protein degradation (Perco et al., 2010). It may also be attributed to the lower abundance of differently expressed proteins which is not detectable by 2D gel electrophoresis compared with microarray analysis. It has been indicated that genes associated with pathways of JA signaling, SA signaling and histone modifications were involved in *trans*-generational priming against biotic stresses (a review, see Balmer et al., 2015). Similarly, a number of genes related to the JA signaling and histone modification pathways were also associated with the *trans*-generational priming against high temperature stress in this study. However, this study did not involve priming in biotic stress and we don’t know if the JA and histone modifications pathways were commonly involved in *trans*-generational priming under both abiotic and biotic stresses, which may be a topic of research in the future.

## Conclusion

**FIGURE 3:**
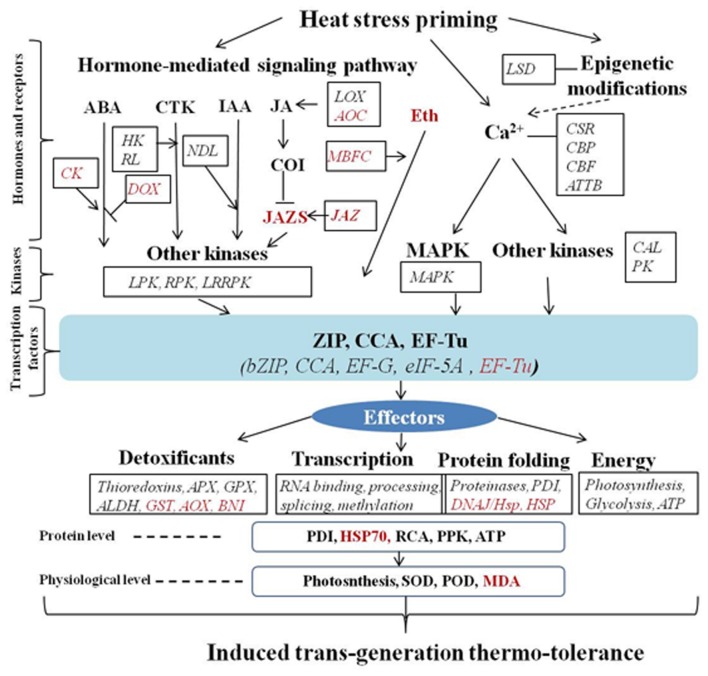
**The proposed mechanism of *trans*-generational tolerance to the high-temperature stress during grain filling in the progeny of primed plants.** The transcriptome physiological analysis suggested that the induced heritable thermo-tolerance by priming the parent generation could activate the processes of epigenetic processes and signaling transduction pathways, which further triggered the modifications of stress defense related processes of defense, transcription, energy, and protein destination and storage in the progeny of primed plants under the reoccurring high-temperature stress. At the proteome level, protein abundance of photosynthesis and energy, and PDI were also up-regulated in the progeny of primed plants compared to non-primed plants in responding to the high-temperature stress, which led to the better maintenance of leaf photosynthesis and activities of photosynthesis and anti-oxidation enzymes, lowered cell membrane damage level and improved heat stress tolerance in progeny of primed plants (italic red means down-regulated, italic black means up-regulated). ALDH, aldehyde dehydrogenase; AOC, allene oxide cyclase; APX: ascorbate POD; ATTB, aldolase-type TIM barrel family protein; bZIP, basic-leucine zipper transcription factor family protein; CAL, concanavalin A-like lectin protein kinase family protein; CBF, calcium-binding EF hand family protein; CBP, Ca^2+^-binding protein; CCA, circadian clock associated 1; CK, casein kinase 1-like; CSR, calcium sensing receptor; DOX, alpha dioxygenase; EF-G, translation elongation factor EFG/EF2 protein; EF-Tu, elongation factor Tu family protein; eIF-5A, eukaryotic elongation factor 5A-3; GPX, glutathione POD; GST, glutathione *S*-transferase family protein; HK, histidine kinase; JAZ, jasmonate-zim-domain protein 1; LOX, lipoxygenase; LPK, lectin protein kinase family protein; LRRPK, leucine-rich receptor-like protein kinase family protein; LSD, lysine-specific histone demethylase 1; MBFC, multiprotein bridging factor 1C; NDL, N-MYC downregulated-like 1; PDI, pyridine nucleotide-disulphide oxidoreductase family protein; PK, protein kinase superfamily protein; PRK, phosphoribulokinase; RA, rubisco activase; RL, ribosomal protein L6 family; RPK, receptor like protein kinase.

In the present study, we observed that thermo-tolerance induced by heat priming during the parental generation could be passed on to the progeny plants to more effectively respond to the successive generation high-temperature stress during grain filling in wheat. This can be exemplified by less grain yield loss, better maintenance of leaf photosynthesis, higher activities of anti-oxidation enzymes, lower cell membrane damage, in the progeny of primed plants than in the progeny of the non-primed plants. The heritable thermo-tolerance induced by priming of the parent generation could activate epigenetic processes and signaling transduction, and trigger modification of stress defense related processes, including the defense, transcription, energy, and protein destination and storage in the progeny of primed plants under high-temperature stress. *Trans*-generation thermo-tolerance was induced by heat priming in the first generation, and this might be an effective measure to cope with severe high-temperature stresses during key growth stages in wheat production. The supposed mechanisms are shown in **Figure 3**.

## Author Contributions

DJ and QZ designed the experiment. XW and CX performed the experiments, analyzed the data and wrote the manuscript. JC, TD, WC, QZ, and DJ involved in the results discussion and editing the manuscript. All authors read and approved the final manuscript.

## Conflict of Interest Statement

The authors declare that the research was conducted in the absence of any commercial or financial relationships that could be construed as a potential conflict of interest.

## Footnotes

1 http://matrixscience.com

2 http://www.ncbi.nlm.nih.gov/

## Abbreviations

A_sat_: Saturated net photosynthesis rate
J_max_: The rate of electron transport
MDA: Malonildialdehyde
POD: Peroxidase
SOD: Superoxide dismutase
V_max_: Maximum carboxylation rate allowed by Rubisco
