# Supplementary material for: Heat Priming Induces Trans-generational Tolerance to High Temperature Stress in Wheat
Source: Front Plant Sci. 2016 Apr 14;7:501. doi: 10.3389/fpls.2016.00501 (PMC4830833; doi:10.3389/fpls.2016.00501)
Supplement: Supplementary file 1 [file Data_Sheet_1.DOC]

**Legends of supplementary figures**

Figure S1 Hierarchical cluster analysis of the expressed genes

Notes: The color of the line shows the expression level of the gene relative to the median in a specific sample: higher expressions in red, lower expressions in green. NC, progeny of non-primed plants without post-anthesis high temperature stress; NH, progeny of non-primed plants with post-anthesis high temperature stress; PC, progeny of primed plants without post-anthesis high temperature stress; and PH, progeny of primed plants with post-anthesis high temperature stress.

Figure S2 **Venn diagrams of proteins which were differently expressed in leaves of the** progenies of primed plants (PH) and non-primed plants (NH) in relation to control (NC) under high-temperature stress during grain filling

Figure S3 Representative 2D gel of progeny of leaf proteins under high-temperature stress during grain filling. The spots numbers indicated by arrows were listed in Table 2.


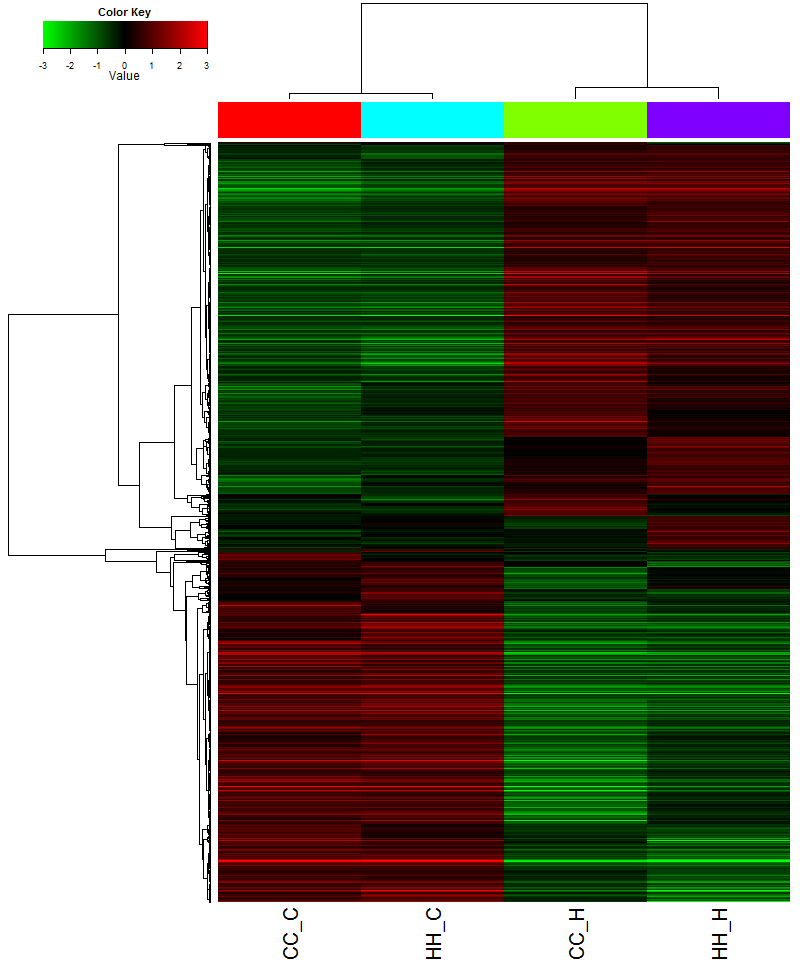


NC PC NH PH


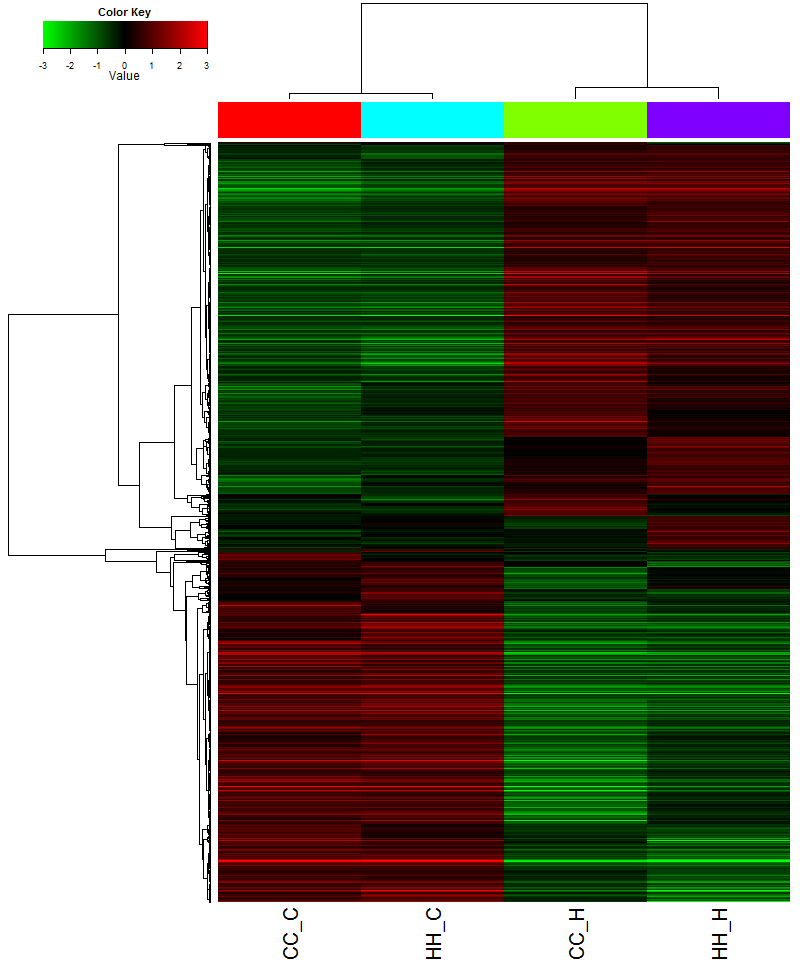


**Figure S1**

**Up- regulated**

**Down- regulated**

**PH/NC**

**NH/NC**

**PH/NC**

**NH/NC**


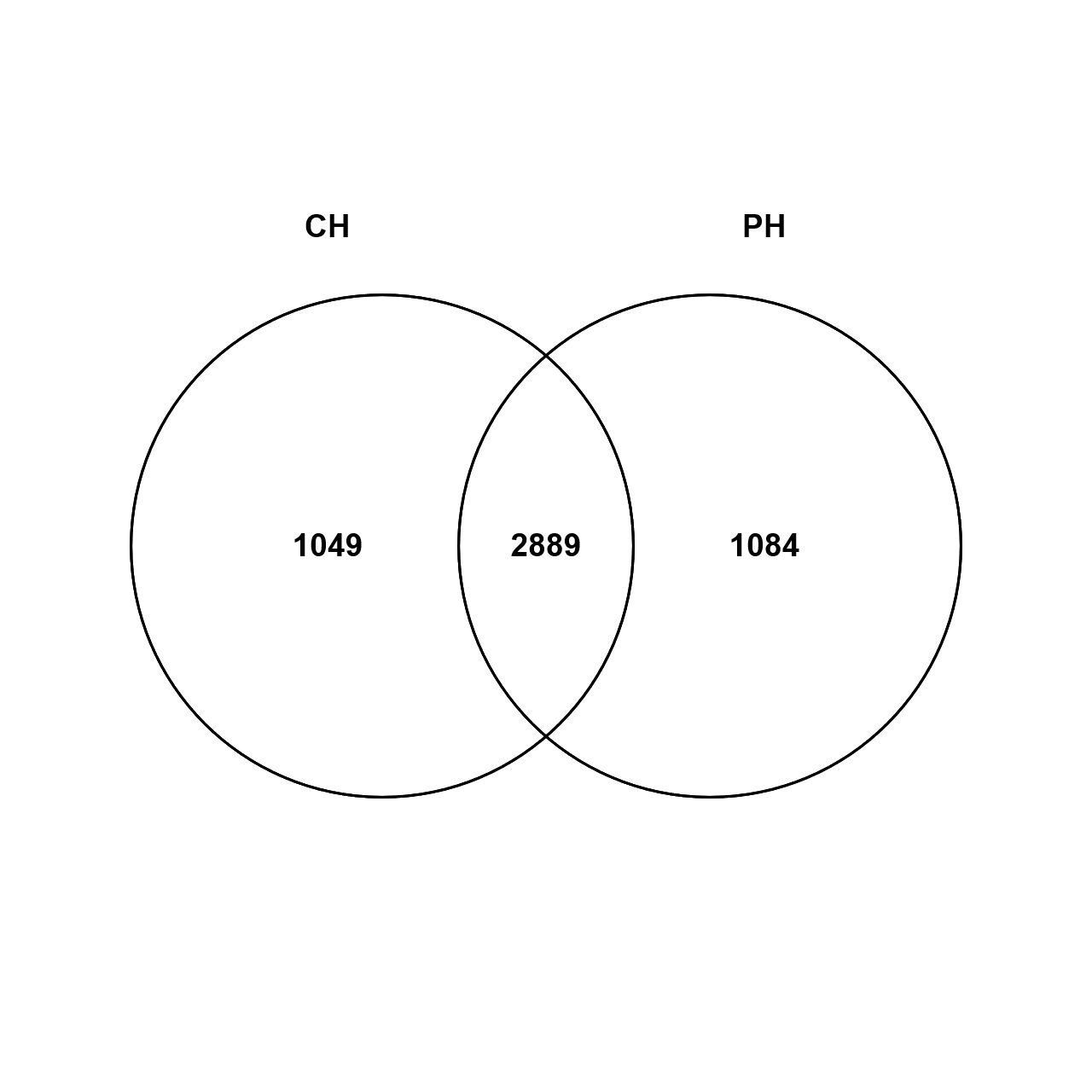

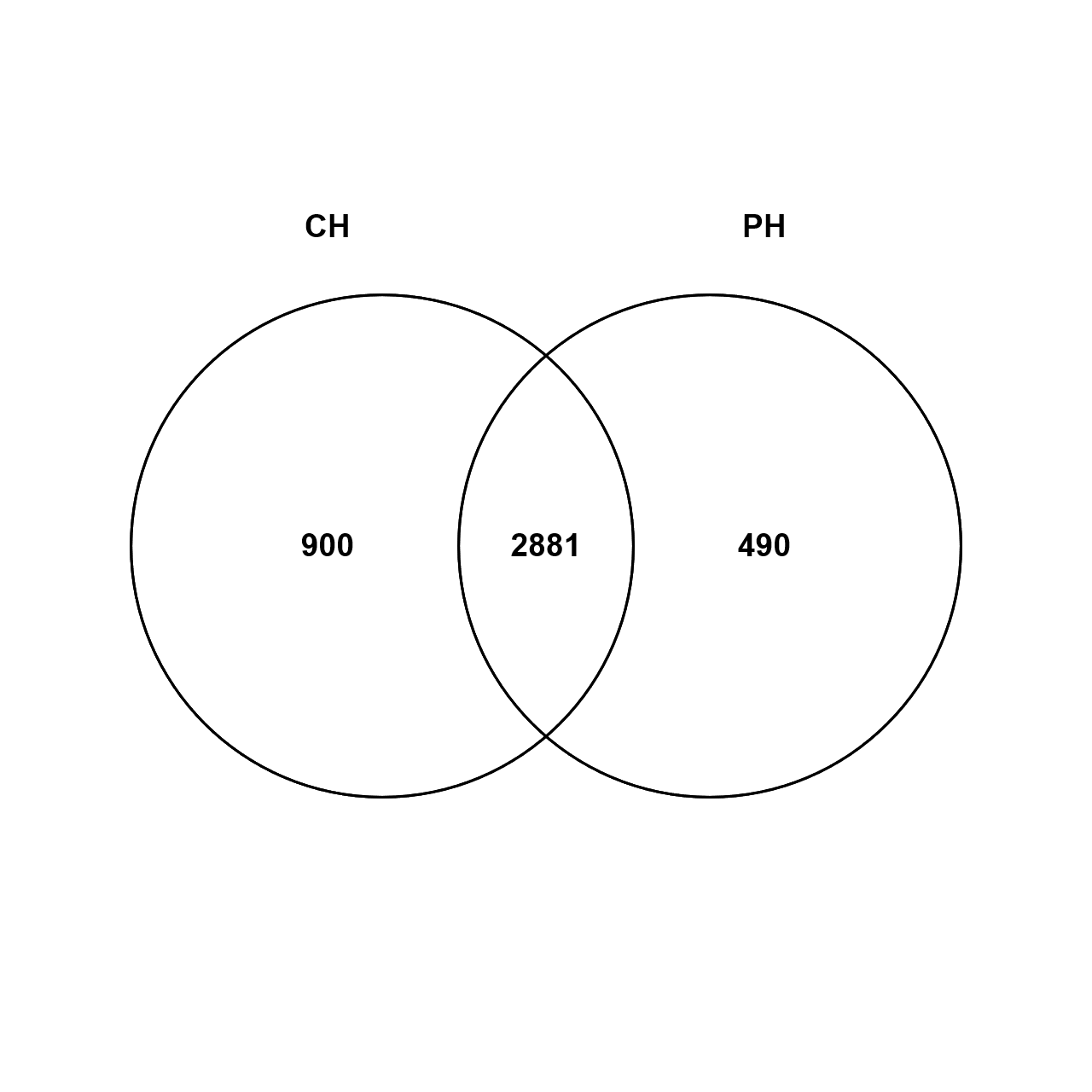


**Figure S2**


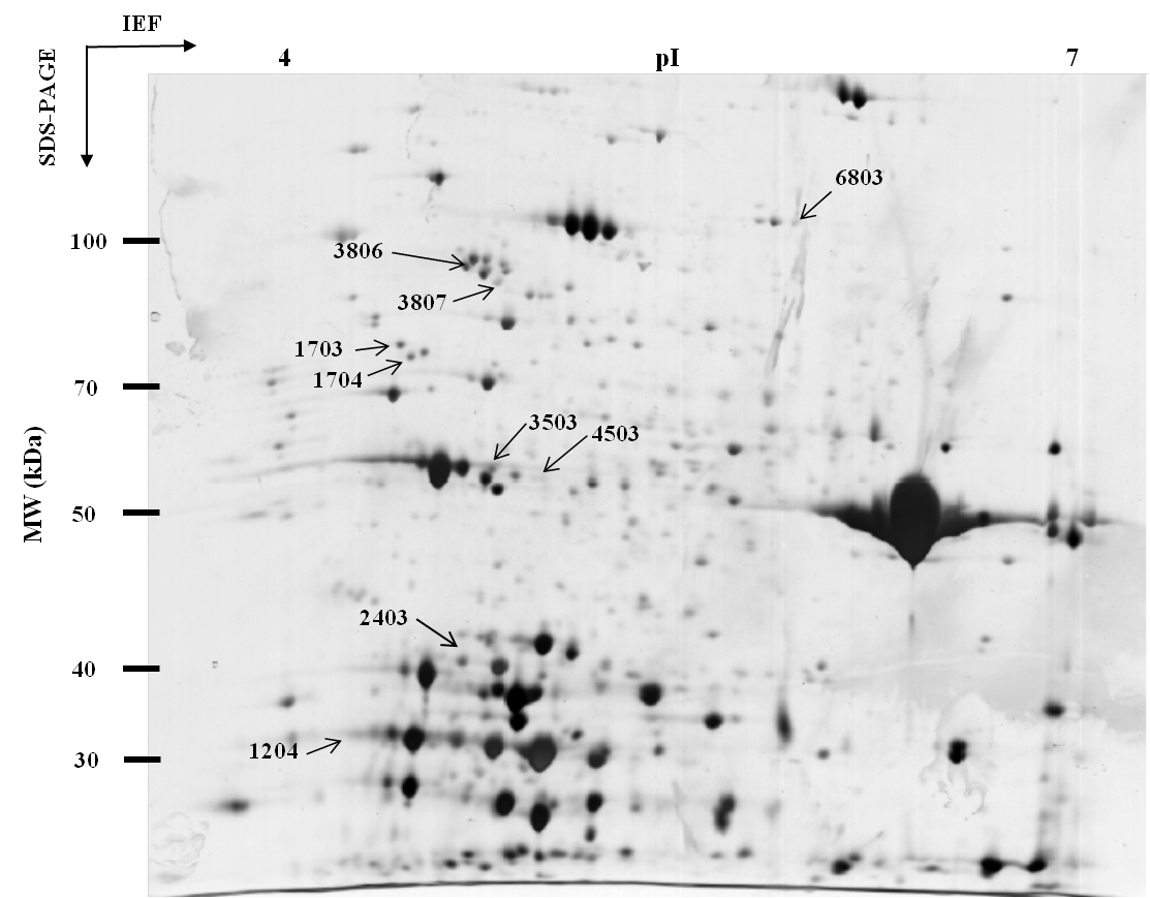


Figure S3
