# Supplementary material for: Heat Priming Induces Trans-generational Tolerance to High Temperature Stress in Wheat
Source: Front Plant Sci. 2016 Apr 14;7:501. doi: 10.3389/fpls.2016.00501 (PMC4830833; doi:10.3389/fpls.2016.00501)
Supplement: Supplementary file 2 [file Data_Sheet_2.DOCX]

Table S1 Genes differently expressed between the progenies of primed plants (PH) and non-primed plants (NH) under high-temperature during grain filling

| **ProbeSetID** | **Ratio** | **GeneDescription** | **Function** |
| --- | --- | --- | --- |
| Ta.581.1.S1_at | 2.6 | Putative acetyl-CoA carboxylase biotin-containing subunit | Metabolism |
| Ta.581.2.S1_a_at | 4.9 | Putative acetyl-CoA carboxylase biotin-containing subunit | Metabolism |
| Ta.24667.1.S1_s_at | 2.1 | GDSL-like Lipase/Acylhydrolase superfamily protein | Metabolism |
| Ta.7943.1.S1_at | 2.5 | Cyclopropane-fatty-acyl-phospholipid synthase | Metabolism |
| Ta.514.1.A1_at | 2.5 | 3-oxo-5-alpha-steroid 4-dehydrogenase family protein | Metabolism |
| TaAffx.111900.1.S1_at | 2.3 | Glycerophosphodiester phosphodiesterase GDPD1 | Metabolism |
| TaAffx.104898.1.S1_at | 2.0 | Glucose-6-phosphate dehydrogenase 1 | Metabolism |
| TaAffx.105423.1.S1_at | 2.2 | Glutamine synthetase 2 | Metabolism |
| TaAffx.37536.1.S1_at | 2.2 | ADC synthase superfamily protein | Metabolism |
| Ta.30505.1.S1_at | 2.6 | Glycine cleavage T-protein family | Metabolism |
| Ta.30795.1.S1_at | 2.4 | Glycine decarboxylase P-protein 2 | Metabolism |
| TaAffx.123343.3.S1_at | 2.2 | Alpha/beta-Hydrolases superfamily protein | Metabolism |
| TaAffx.14510.1.S1_at | 2.7 | Alpha/beta-Hydrolases superfamily protein | Metabolism |
| Ta.25039.2.S1_x_at | 2.5 | Alpha/beta-Hydrolases superfamily protein | Metabolism |
| Ta.25039.2.S1_at | 2.3 | Alpha/beta-Hydrolases superfamily protein | Metabolism |
| TaAffx.58649.1.S1_at | 2.5 | P-loop containing nucleoside triphosphate hydrolases superfamily protein | Metabolism |
| Ta.1166.1.A1_x_at | 3.2 | Inositol monophosphatase family protein | Metabolism |
| Ta.1166.2.S1_a_at | 2.3 | Inositol monophosphatase family protein | Metabolism |
| Ta.1117.1.S1_at | 2.0 | Alanine-2-oxoglutarate aminotransferase 2 | Metabolism |
| Ta.1426.1.S1_x_at | 7.4 | PsbQ-like 2 | Energy |
| Ta.1426.3.S1_s_at | 12.0 | PsbQ-like 2 | Energy |
| Ta.1139.1.S1_at | 3.5 | Light harvesting complex of photosystem II 5 | Energy |
| Ta.1139.1.S1_x_at | 3.4 | Light harvesting complex of photosystem II 5 | Energy |
| Ta.25600.1.S1_x_at | 2.7 | Light harvesting complex photosystem II subunit 6 | Energy |
| Ta.22984.2.S1_x_at | 2.0 | Light-harvesting chlorophyll-protein complex II subunit B1 | Energy |
| Ta.20639.1.S1_x_at | 2.8 | Light-harvesting chlorophyll-protein complex I subunit A4 | Energy |
| Ta.20639.2.A1_a_at | 2.3 | Light-harvesting chlorophyll-protein complex I subunit A4 | Energy |
| Ta.20639.3.S1_a_at | 3.2 | Light-harvesting chlorophyll-protein complex I subunit A4 | Energy |
| Ta.20639.3.S1_x_at | 2.9 | Light-harvesting chlorophyll-protein complex I subunit A4 | Energy |
| Ta.27751.7.A1_x_at | 4.6 | Light-harvesting chlorophyll-protein complex II subunit B1 | Energy |
| Ta.3795.1.S1_x_at | 2.2 | Light-harvesting chlorophyll-protein complex II subunit B1 | Energy |
| Ta.881.2.S1_x_at | 2.0 | Photosystem I light harvesting complex gene 5 | Energy |
| Ta.2402.3.S1_x_at | 2.7 | Photosystem I light harvesting complex gene 1 | Energy |
| Ta.12065.1.S1_at | 2.4 | NDH-dependent cyclic electron flow 1 | Energy |
| Ta.981.1.S1_a_at | 2.1 | NDH-dependent cyclic electron flow 1 | Energy |
| Ta.981.1.S1_at | 2.1 | NDH-dependent cyclic electron flow 1 | Energy |
| Ta.981.1.S1_x_at | 2.0 | NDH-dependent cyclic electron flow 1 | Energy |
| Ta.981.2.S1_at | 2.8 | NDH-dependent cyclic electron flow 1 | Energy |
| Ta.9368.2.S1_x_at | 3.1 | NDH-dependent cyclic electron flow 6 | Energy |
| Ta.1988.1.S1_x_at | 2.2 | Sedoheptulose-bisphosphatase | Energy |
| Ta.1988.2.S1_x_at | 2.2 | Sedoheptulose-bisphosphatase | Energy |
| Ta.1988.3.S1_x_at | 2.2 | Sedoheptulose-bisphosphatase | Energy |
| Ta.20429.2.S1_at | 3.0 | Aldolase superfamily protein | Energy |
| Ta.447.1.S1_x_at | 6.4 | Aldolase superfamily protein | Energy |
| Ta.447.2.S1_a_at | 12.7 | Aldolase superfamily protein | Energy |
| Ta.3916.1.S1_a_at | 2.2 | 2Fe-2S ferredoxin-like superfamily protein | Energy |
| Ta.3916.2.S1_x_at | 2.5 | 2Fe-2S ferredoxin-like superfamily protein | Energy |
| Ta.2752.2.S1_x_at | 3.0 | Ribulose bisphosphate carboxylase small chain 1A | Energy |
| Ta.2752.3.S1_x_at | 2.3 | Ribulose bisphosphate carboxylase small chain 1A | Energy |
| Ta.27923.2.S1_x_at | 2.1 | Ribulose bisphosphate carboxylase small chain 1A | Energy |
| TaAffx.108219.1.S1_at | 4.0 | Ribulose bisphosphate carboxylase small chain 1A | Energy |
| TaAffx.449.1.A1_at | 3.6 | Ribulose bisphosphate carboxylase small chain 1A | Energy |
| Ta.27646.1.S1_at | 3.8 | Photosystem I light harvesting complex gene 3 | Energy |
| Ta.27646.1.S1_x_at | 3.8 | Photosystem I light harvesting complex gene 3 | Energy |
| Ta.28363.2.S1_a_at | 2.4 | Photosystem I reaction center subunit PSI-N, chloroplast | Energy |
| Ta.27751.2.S1_x_at | 3.5 | Photosystem II light harvesting complex gene 2.3 | Energy |
| Ta.27751.6.S1_at | 2.6 | Photosystem II light harvesting complex gene 2.2 | Energy |
| TaAffx.128506.1.S1_x_at | 2.3 | Photosystem II light harvesting complex gene 2.3 | Energy |
| Ta.8061.1.S1_at | 3.7 | Photosystem II reaction center PSB28 protein | Energy |
| Ta.27761.1.S1_x_at | 4.5 | Photosystem I subunit K | Energy |
| Ta.27761.2.S1_x_at | 4.8 | Photosystem I subunit K | Energy |
| Ta.27761.3.S1_x_at | 4.4 | Photosystem I subunit K | Energy |
| Ta.3581.1.S1_x_at | 2.1 | Photosystem I subunit F | Energy |
| Ta.3581.3.S1_x_at | 2.8 | Photosystem I subunit F | Energy |
| Ta.1167.1.S1_at | 2.2 | Ferredoxin-NADP(+)-oxidoreductase 1 | Energy |
| Ta.28806.1.S1_at | 3.5 | Ferredoxin-NADP(+)-oxidoreductase 1 | Energy |
| TaAffx.128473.1.A1_s_at | 3.1 | ATP synthase alpha/beta family protein | Energy |
| TaAffx.27201.1.S1_at | 3.0 | ATP synthase alpha/beta family protein | Energy |
| Ta.23120.1.S1_at | 2.2 | ATPase gamma subunit protein | Energy |
| Ta.25528.2.S1_at | 2.5 | Starch synthase 3 | Energy |
| Ta.30808.1.S1_s_at | 2.8 | Glyceraldehyde 3-phosphate dehydrogenase A subunit 2 | Energy |
| Ta.1135.1.S1_at | 2.1 | Glyceraldehyde-3-phosphate dehydrogenase B subunit | Energy |
| Ta.12118.1.S1_a_at | 2.9 | Beta glucosidase 10 | Energy |
| TaAffx.11282.1.A1_at | 3.6 | Beta glucosidase 8 | Energy |
| Ta.4124.1.A1_at | 2.9 | Phosphofructokinase 2 | Energy |
| Ta.4637.1.A1_at | 2.1 | Phosphofructokinase 7 | Energy |
| Ta.1161.1.S1_at | 4.6 | Chlorophyll A-B binding family protein | Energy |
| Ta.25440.1.S1_a_at | 2.6 | Chloroplast thylakoid lumen protein | Energy |
| TaAffx.12573.1.S1_at | 2.2 | Thylakoid lumenal 17.9 kDa protein, chloroplast | Energy |
| Ta.7005.2.S1_at | 2.4 | Galactose mutarotase-like superfamily protein | Energy |
| Ta.7005.3.S1_a_at | 2.1 | Galactose mutarotase-like superfamily protein | Energy |
| Ta.5227.1.S1_at | 2.2 | Carbonic anhydrase 2 | Energy |
| Ta.5227.1.S1_x_at | 2.6 | Carbonic anhydrase 2 | Energy |
| Ta.2713.2.S1_a_at | 3.6 | Plastid-lipid associated protein PAP / fibrillin family protein | Energy |
| Ta.5064.1.A1_at | 2.0 | Phosphoglycerate mutase-like family protein | Energy |
| Ta.23158.1.S1_at | 2.7 | Phosphoribulokinase | Energy |
| Ta.2392.1.S1_x_at | 2.4 | Cytochrome b6f complex subunit | Energy |
| Ta.23974.1.S1_at | 2.4 | FK506-binding protein 16-2 | Energy |
| Ta.7769.1.A1_at | 3.1 | Rieske (2Fe-2S) domain-containing protein | Energy |
| Ta.3256.1.S1_at | 5.6 | 2-phosphoglycolate phosphatase 1 | Energy |
| Ta.439.1.S1_at | 2.8 | High cyclic electron flow 1 | Energy |
| TaAffx.11961.1.S1_at | 2.3 | Haloacid dehalogenase-like hydrolase family protein | Energy |
| TaAffx.32199.1.S1_at | 2.9 | Phosphoglucomutase, putative / glucose phosphomutase, putative | Energy |
| TaAffx.38244.1.S1_at | 2.2 | UDP-glucose pyrophosphorylase 3 | Energy |
| TaAffx.50825.1.S1_at | 2.2 | DEAD/DEAH box helicase, putative | Energy |
| Ta.20776.1.S1_at | 2.1 | Phytoene synthase | Energy |
| Ta.3788.1.A1_at | 2.1 | Translocon at the inner envelope membrane of chloroplasts 110 | Energy |
| Ta.21208.1.S1_at | 4.0 | Ferric reduction oxidase 6 | Energy |
| TaAffx.92581.1.S1_at | 3.5 | UDP- glucosyltransferases | Energy |
| Ta.2307.1.S1_at | 2.1 | Phosphoglycerate kinase family protein | Energy |
| Ta.650.1.A1_at | 2.8 | Xylulose kinase-1 | Energy |
| Ta.12952.2.S1_at | 2.0 | PfkB-like carbohydrate kinase family protein | Energy |
| Ta.12952.3.S1_s_at | 2.4 | PfkB-like carbohydrate kinase family protein | Energy |
| Ta.769.1.S1_at | 2.6 | Plastid transcriptionally active 16 | Energy |
| TaAffx.31811.1.S1_s_at | 2.1 | Flavodoxin family protein | Energy |
| Ta.12151.1.A1_at | 2.4 | DNA mismatch repair protein MutS, type 2 | Cell growth |
| TaAffx.12595.1.S1_at | 2.5 | DNA gyrase B1 | Cell growth |
| TaAffx.13379.1.S1_s_at | 2.3 | Actin binding | Cell growth |
| TaAffx.44042.1.S1_x_at | 2.1 | Tetraspanin family protein | Cell growth |
| TaAffx.98080.1.A1_s_at | 2.1 | Restriction endonuclease, type II-like superfamily protein | Cell growth |
| Ta.26907.1.S1_at | 5.2 | Ribonuclease T2 family protein | Transcription |
| Ta.22762.2.S1_at | 2.1 | Chloroplast stem-loop binding protein of 41 kDa | Transcription |
| TaAffx.15128.1.S1_at | 3.2 | Ribosomal protein L24 | Transcription |
| TaAffx.74362.1.S1_at | 2.0 | Binding to TOMV RNA 1L (long form) | Transcription |
| TaAffx.7500.1.S1_at | 2.9 | KH domain-containing protein | Transcription |
| Ta.1983.1.S1_at | 4.8 | Chloroplast RNA binding | Transcription |
| Ta.13215.1.S1_at | 2.3 | Ribonucleotide reductase 1 | Transcription |
| Ta.8299.1.S1_at | 2.2 | U2 small nuclear ribonucleoprotein A | Transcription |
| Ta.6762.1.A1_at | 2.0 | NOP56-like pre RNA processing ribonucleoprotein | Transcription |
| TaAffx.45318.1.S1_s_at | 2.1 | D111/G-patch domain-containing protein | Transcription |
| TaAffx.93007.1.S1_at | 2.2 | RNA-binding family protein | Transcription |
| Ta.15829.1.S1_at | 2.0 | Arginyl-tRNA synthetase, class Ic | Transcription |
| Ta.491.2.S1_at | 2.2 | Putative mitochondrial RNA helicase 1 | Transcription |
| TaAffx.64230.1.S1_at | 2.4 | Uridylyltransferase-related | Transcription |
| TaAffx.86186.1.S1_at | 4.3 | FtsJ-like methyltransferase family protein | Transcription |
| Ta.12172.1.S1_at | 5.7 | S-adenosyl-L-methionine-dependent methyltransferases superfamily protein | Transcription |
| Ta.12212.1.S1_at | 2.7 | S-adenosyl-L-methionine-dependent methyltransferases superfamily protein | Transcription |
| Ta.6594.1.S1_at | 2.5 | Lysine-specific histone demethylase 1 homolog 1 | Transcription |
| Ta.28069.1.A1_at | 2.4 | Lysine-specific histone demethylase 1 homolog 1 | Transcription |
| Ta.5948.1.S1_at | 2.3 | Lysine-specific histone demethylase 1 homolog 1 | Transcription |
| Ta.6812.1.S1_at | 2.1 | Basic-leucine zipper (bZIP) transcription factor family protein | Protein synthesis |
| Ta.7524.2.A1_x_at | 2.4 | Circadian clock associated 1 | Protein synthesis |
| Ta.28228.1.S1_at | 2.6 | Translation elongation factor EFG/EF2 protein | Protein synthesis |
| Ta.28228.2.S1_at | 2.4 | Translation elongation factor EFG/EF2 protein | Protein synthesis |
| TaAffx.129719.1.S1_s_at | 2.7 | Translation elongation factor EFG/EF2 protein | Protein synthesis |
| Ta.23277.1.S1_a_at | 2.4 | SMAD/FHA domain-containing protein | Protein synthesis |
| TaAffx.9432.1.A1_at | 2.5 | Eukaryotic elongation factor 5A-3 | Protein synthesis |
| Ta.1151.1.S1_at | 3.6 | Cysteine proteinases superfamily protein | Protein destination and storage |
| Ta.191.1.S1_at | 3.2 | Cysteine proteinases superfamily protein | Protein destination and storage |
| Ta.4651.1.S1_at | 2.4 | Eukaryotic aspartyl protease family protein | Protein destination and storage |
| Ta.537.1.A1_at | 2.1 | CAAX amino terminal protease family protein | Protein destination and storage |
| TaAffx.13302.1.S1_at | 2.0 | ATP-dependent protease La (LON) domain protein | Protein destination and storage |
| Ta.27038.1.S1_at | 2.1 | PDI-like 1-4 | Protein destination and storage |
| TaAffx.92900.1.S1_at | 2.1 | TCP-1/cpn60 chaperonin family protein | Protein destination and storage |
| Ta.3756.1.S1_x_at | 2.1 | Cyclophilin 38 | Protein destination and storage |
| Ta.1574.1.S1_s_at | 2.6 | Copper chaperone | Transporters |
| Ta.18466.1.S1_at | 2.2 | Amino acid permease 1 | Transporters |
| Ta.12133.1.A1_at | 2.3 | MATE efflux family protein | Transporters |
| Ta.862.1.A1_at | 2.2 | Phosphoenolpyruvate (pep)/phosphate translocator 2 | Transporters |
| TaAffx.104904.1.S1_x_at | 2.2 | CHY-type/CTCHY-type/RING-type Zinc finger protein | Transporters |
| TaAffx.105340.1.S1_at | 2.2 | Voltage-gated chloride channel family protein | Transporters |
| TaAffx.113857.2.S1_s_at | 2.4 | Major facilitator superfamily protein | Transporters |
| TaAffx.12039.1.S1_at | 2.6 | ABC transporter B family member 21 | Transporters |
| TaAffx.120297.1.S1_at | 2.2 | ABC transporter C family member 9 | Transporters |
| Ta.28087.1.S1_s_at | 2.7 | Sec14p-like phosphatidylinositol transfer family protein | Transporters |
| Ta.4001.1.S1_at | 5.4 | Phosphate transporter 2;1 | Transporters |
| Ta.429.1.S1_at | 2.2 | Farnesyl diphosphate synthase 1 | Transporters |
| TaAffx.55592.1.S1_at | 2.0 | High affinity K^+^ transporter 5 | Transporters |
| Ta.21321.1.S1_s_at | 2.2 | Divalent ion symporter | Transporters |
| TaAffx.80863.1.S1_at | 2.4 | Aminopeptidase M1 | Transporters |
| Ta.1848.2.S1_x_at | 2.3 | Plasma membrane intrinsic protein 1-4 | Transporters |
| TaAffx.21600.1.S1_s_at | 2.0 | Mitochondrial substrate carrier family protein | Cell structure |
| TaAffx.21600.2.S1_x_at | 2.0 | Mitochondrial substrate carrier family protein | Cell structure |
| Ta.23293.1.S1_at | 2.4 | Peroxisomal membrane 22 kDa (Mpv17/PMP22) family protein | Cell structure |
| Ta.23293.1.S1_x_at | 2.3 | Peroxisomal membrane 22 kDa (Mpv17/PMP22) family protein | Cell structure |
| Ta.8201.1.S1_at | 2.2 | Peroxisomal membrane 22 kDa (Mpv17/PMP22) family protein | Cell structure |
| Ta.9308.1.S1_x_at | 2.1 | Peroxin 11c | Cell structure |
| Ta.9308.2.S1_x_at | 2.6 | Peroxin 11c | Cell structure |
| TaAffx.84810.1.S1_x_at | 2.0 | Peroxin 11c | Cell structure |
| Ta.3721.1.S1_x_at | 2.1 | Leucine-rich repeat (LRR) family protein | Cell structure |
| Ta.1279.1.S1_at | 2.1 | Glycosyl transferase, family 35 | Cell structure |
| Ta.20195.1.S1_x_at | 2.2 | Proline-rich extensin-like family protein | Cell structure |
| TaAffx.33840.1.A1_at | 2.2 | MAP kinase 20 | Signal transduction |
| Ta.29561.1.A1_at | 2.4 | Protein kinase superfamily protein | Signal transduction |
| TaAffx.81369.1.S1_at | 2.3 | Concanavalin A-like lectin protein kinase family protein | Signal transduction |
| Ta.3943.1.A1_at | 2.2 | Membrane protein, putative | Signal transduction |
| Ta.25836.1.S1_at | 3.1 | Calcium-binding EF hand family protein | Signal transduction |
| Ta.2381.1.S1_at | 2.7 | Calcium sensing receptor | Signal transduction |
| Ta.5511.1.S1_at | 2.5 | Ca^2+^-binding protein 1 | Signal transduction |
| Ta.28197.1.S1_at | 2.6 | Aldolase-type TIM barrel family protein | Signal transduction |
| Ta.25825.1.A1_at | 2.2 | S-domain-2 (SD2) receptor kinase 5 | Signal transduction |
| TaAffx.29269.1.S1_at | 2.1 | S-locus lectin protein kinase family protein | Signal transduction |
| TaAffx.56334.2.S1_at | 2.5 | Casein kinase 1-like protein 2 | Signal transduction |
| TaAffx.92089.1.S1_at | 2.4 | Casein kinase 1-like protein 2 | Signal transduction |
| TaAffx.66712.1.S1_at | 2.2 | Lectin protein kinase family protein | Signal transduction |
| Ta.485.1.A1_at | 2.6 | Lipoxygenase 2 | Signal transduction |
| Ta.12127.1.A1_at | 3.4 | Leucine-rich receptor-like protein kinase family protein | Signal transduction |
| TaAffx.110753.1.S1_at | 2.1 | Leucine-rich repeat protein kinase family protein | Signal transduction |
| TaAffx.23369.2.S1_at | 2.3 | Leucine-rich repeat protein kinase family protein | Signal transduction |
| TaAffx.80605.1.S1_at | 2.2 | Leucine-rich repeat transmembrane protein kinase | Signal transduction |
| TaAffx.4501.4.S1_s_at | 4.3 | Receptor like protein kinase 32 | Signal transduction |
| TaAffx.83756.1.S1_at | 2.0 | Phytosulfokin receptor 1 | Signal transduction |
| Ta.963.2.A1_at | 2.0 | Histidine kinase 3 | Signal transduction |
| Ta.6537.2.A1_a_at | 2.6 | Ribosomal protein L6 family | Signal transduction |
| TaAffx.109116.1.S1_at | 2.7 | Receptor-like protein kinase 4 | Signal transduction |
| Ta.11303.1.A1_at | 2.3 | N-MYC downregulated-like 1 | Signal transduction |
| Ta.1364.1.S1_at | 2.2 | Pyridine nucleotide-disulphide oxidoreductase family protein | Defense |
| Ta.12341.1.S1_at | 2.0 | Thioredoxin superfamily protein | Defense |
| Ta.23137.1.S1_at | 2.1 | Thioredoxin family protein | Defense |
| Ta.13016.1.S1_at | 2.2 | FAD/NAD(P)-binding oxidoreductase family protein | Defense |
| Ta.511.1.S1_at | 3.3 | Aldehyde dehydrogenase 11A3 | Defense |
| Ta.488.1.S1_at | 2.8 | Ascorbate peroxidase 4 | Defense |
| Ta.488.1.S1_x_at | 2.8 | Ascorbate peroxidase 4 | Defense |
| TaAffx.21646.1.S1_at | 2.9 | Peroxidase superfamily protein | Defense |
| TaAffx.32266.1.A1_at | 3.2 | Glutathione peroxidase 6 | Defense |
| Ta.1138.1.S1_at | 2.1 | Late embryogenesis abundant protein (LEA) family protein | Defense |
| TaAffx.15425.1.A1_at | 3.0 | Drought-responsive family protein | Defense |
| Ta.7213.2.S1_at | 2.3 | Putative GTP diphosphokinase RSH1 | Defense |
| Ta.27135.1.S1_at | 3.3 | Serine transhydroxymethyltransferase 1 | Defense |
| Ta.22286.1.S1_at | 2.8 | Tropinone reductase | Secondary Metabolism |
| Ta.27726.1.S1_at | 2.6 | Cinnamyl alcohol dehydrogenase 6 | Secondary Metabolism |
| Ta.29583.1.S1_at | 2.4 | One helix protein | Unclear |
| TaAffx.66109.1.S1_at | 2.3 | Ribonuclease II/R family protein | Unclear |
| Ta.24045.1.A1_x_at | 2.3 | Haloacid dehalogenase-like hydrolase (HAD) superfamily protein | Unclear |
| Ta.2431.1.S1_at | 2.3 | Haloacid dehalogenase-like hydrolase (HAD) superfamily protein | Unclear |
| Ta.2431.1.S1_x_at | 2.2 | Haloacid dehalogenase-like hydrolase (HAD) superfamily protein | Unclear |
| Ta.2431.2.S1_x_at | 2.6 | Haloacid dehalogenase-like hydrolase (HAD) superfamily protein | Unclear |
| TaAffx.13031.1.S1_at | 2.1 | ARM repeat superfamily protein | Unclear |
| Ta.10389.1.S1_a_at | 2.1 | NAD(P)-binding Rossmann-fold superfamily protein | Unclear |
| Ta.17258.1.S1_at | 2.1 | NAD(P)-binding Rossmann-fold superfamily protein | Unclear |
| Down regulated |  |  |  |
| TaAffx.79139.1.S1_at | 0.30 | Isocitrate lyase | Metabolism |
| TaAffx.79218.1.S1_at | 0.32 | Isocitrate lyase | Metabolism |
| TaAffx.99992.1.S1_at | 0.28 | Isocitrate lyase | Metabolism |
| Ta.23712.1.S1_at | 0.48 | Acyl-CoA binding protein 4 | Metabolism |
| Ta.24254.1.S1_a_at | 0.49 | Fatty acid desaturase 7 | Metabolism |
| Ta.12996.2.S1_at | 0.46 | Cytosolic NADP+-dependent isocitrate dehydrogenase | Metabolism |
| Ta.1906.1.S1_at | 0.43 | Class-II DAHP synthetase family protein | Metabolism |
| Ta.2631.3.S1_x_at | 0.44 | Myo-inositol oxygenase 5 | Metabolism |
| Ta.27457.5.S1_x_at | 0.39 | Metallothionein 2B | Metabolism |
| Ta.30758.1.S1_at | 0.48 | Glycine-rich protein family | Metabolism |
| Ta.9122.1.S1_at | 0.48 | Arogenate dehydratase 6 | Metabolism |
| Ta.6223.1.S1_at | 0.35 | Glutamine-dependent asparagine synthase 1 | Metabolism |
| TaAffx.97964.1.S1_at | 0.42 | Alpha-amylase-like | Metabolism |
| Ta.2471.3.S1_a_at | 0.30 | P-loop containing nucleoside triphosphate hydrolases superfamily protein | Metabolism |
| Ta.2471.3.S1_x_at | 0.43 | P-loop containing nucleoside triphosphate hydrolases superfamily protein | Metabolism |
| TaAffx.21593.1.S1_at | 0.13 | Pyridoxal phosphate (PLP)-dependent transferases superfamily protein | Metabolism |
| Ta.1258.2.S1_x_at | 0.37 | S-adenosyl-L-homocysteine hydrolase | Metabolism |
| Ta.22718.1.S1_at | 0.36 | Alpha/beta-Hydrolases superfamily protein | Metabolism |
| Ta.23112.1.S1_x_at | 0.15 | Methionine gamma-lyase | Metabolism |
| Ta.22452.1.S1_at | 0.47 | Azelaic acid induced 1 | Metabolism |
| Ta.225.1.S1_at | 0.17 | Glycosyl hydrolase superfamily protein | Energy |
| Ta.3828.3.A1_a_at | 0.46 | Glycosyl hydrolase superfamily protein | Energy |
| Ta.23909.1.S1_a_at | 0.39 | B-S glucosidase 44 | Energy |
| Ta.23909.2.S1_at | 0.37 | Beta glucosidase 43 | Energy |
| TaAffx.52900.1.S1_at | 0.45 | Alpha carbonic anhydrase 4 | Energy |
| Ta.22537.1.S1_x_at | 0.40 | Alpha carbonic anhydrase 8 | Energy |
| Ta.10680.1.S1_at | 0.50 | Galactose mutarotase-like superfamily protein | Energy |
| Ta.13785.1.S1_at | 0.19 | Chitinase A | Energy |
| Ta.31.1.S1_at | 0.33 | Mannose-binding lectin superfamily protein | Energy |
| Ta.3828.3.A1_x_at | 0.45 | Glycosyl hydrolase superfamily protein | Energy |
| Ta.9208.1.S1_at | 0.47 | Apyrase 2 | Energy |
| Ta.30327.1.S1_at | 0.26 | UDP-Glycosyltransferase superfamily protein | Energy |
| TaAffx.23237.1.S1_at | 0.48 | UDP-Glycosyltransferase superfamily protein | Energy |
| Ta.30327.2.A1_at | 0.30 | UDP-glucosyl transferase 88A1 | Energy |
| Ta.23272.1.S1_at | 0.45 | UDP-glucosyl transferase 73C6 | Energy |
| Ta.2657.1.S1_x_at | 0.16 | UDP-glucose 6-dehydrogenase family protein | Energy |
| TaAffx.68872.1.S1_at | 0.46 | Chloroplast beta-amylase | Energy |
| Ta.24991.1.S1_x_at | 0.47 | Glyceraldehyde-3-phosphate dehydrogenase C subunit 1 | Energy |
| Ta.8782.1.S1_at | 0.46 | Pectin lyase-like superfamily protein | Cell structure |
| Ta.18203.1.S1_at | 0.06 | Cupredoxin superfamily protein | Cell structure |
| TaAffx.107606.1.A1_at | 0.46 | RING membrane-anchor 3 | Cell structure |
| Ta.9765.1.S1_x_at | 0.29 | Cell wall invertase 2 | Cell structure |
| TaAffx.16032.1.A1_at | 0.35 | Cell wall invertase 2 | Cell structure |
| Ta.135.2.S1_at | 0.32 | Reversibly glycosylated polypeptide 1 | Cell structure |
| TaAffx.102561.2.S1_at | 0.42 | RHO guanyl-nucleotide exchange factor 11 | Cell structure |
| Ta.5046.1.S1_at | 0.50 | Expansin A4 | Cell structure |
| TaAffx.3462.1.S1_at | 0.49 | Leucine-rich repeat (LRR) family protein | Cell structure |
| TaAffx.88904.1.S1_x_at | 0.19 | Leucine-rich repeat (LRR) family protein | Cell structure |
| TaAffx.12166.1.A1_at | 0.40 | Wall associated kinase 3 | Cell structure |
| TaAffx.119013.1.S1_at | 0.25 | General regulatory factor 5 | Cell structure |
| Ta.23763.1.S1_at | 0.20 | PLAT/LH2 domain-containing lipoxygenase family protein | Transcription |
| Ta.28171.1.S1_at | 0.50 | PLAT/LH2 domain-containing lipoxygenase family protein | Transcription |
| Ta.9742.2.A1_a_at | 0.48 | PLAT/LH2 domain-containing lipoxygenase family protein | Transcription |
| Ta.6103.1.A1_at | 0.48 | Splicing factor Prp18 family protein | Transcription |
| Ta.7339.1.S1_x_at | 0.19 | K-box region and MADS-box transcription factor family protein | Transcription |
| TaAffx.13990.1.S1_at | 0.43 | RING/U-box superfamily protein | Transcription |
| Ta.9488.3.S1_x_at | 0.45 | CTC-interacting domain 8 | Transcription |
| Ta.4917.1.S1_at | 0.35 | S-adenosyl-L-methionine-dependent methyltransferases superfamily protein | Transcription |
| Ta.5127.2.S1_a_at | 0.46 | NAC domain containing protein 1 | Transcription |
| Ta.5367.2.S1_x_at | 0.47 | NAC domain transcriptional regulator superfamily protein | Transcription |
| Ta.18638.1.S1_at | 0.45 | Xylem NAC domain 1 | Transcription |
| TaAffx.12101.1.S1_at | 0.29 | GTP binding Elongation factor Tu family protein | Protein synthesis |
| TaAffx.80168.1.S1_at | 0.42 | GTP binding Elongation factor Tu family protein | Protein synthesis |
| Ta.10390.1.S1_at | 0.28 | Cysteine proteinases superfamily protein | Protein destination and storage |
| Ta.10390.2.A1_a_at | 0.41 | Cysteine proteinases superfamily protein | Protein destination and storage |
| Ta.10390.2.A1_x_at | 0.31 | Cysteine proteinases superfamily protein | Protein destination and storage |
| Ta.747.1.S1_at | 0.30 | Eukaryotic aspartyl protease family protein | Protein destination and storage |
| TaAffx.21249.1.S1_at | 0.39 | Eukaryotic aspartyl protease family protein | Protein destination and storage |
| TaAffx.21249.1.S1_x_at | 0.35 | Eukaryotic aspartyl protease family protein | Protein destination and storage |
| Ta.22493.1.S1_x_at | 0.47 | Chaperone DnaJ-domain superfamily protein | Protein destination and storage |
| TaAffx.37294.1.S1_at | 0.35 | Chaperone DnaJ-domain superfamily protein | Protein destination and storage |
| Ta.119.1.S1_x_at | 0.30 | Serine protease inhibitor (SERPIN) family protein | Protein destination and storage |
| TaAffx.132498.1.S1_at | 0.46 | Serine protease inhibitor, potato inhibitor I-type family protein | Protein destination and storage |
| Ta.2747.1.S1_at | 0.46 | Heat shock protein 21 | Protein destination and storage |
| Ta.261.1.S1_at | 0.47 | Heat shock protein 101 | Protein destination and storage |
| Ta.10259.1.S1_at | 0.48 | Heat shock protein 70 | Protein destination and storage |
| Ta.204.1.S1_at | 0.50 | HSP20-like chaperones superfamily protein | Protein destination and storage |
| Ta.22973.1.S1_x_at | 0.37 | 17.6 kDa class II heat shock protein | Protein destination and storage |
| Ta.28083.1.S1_at | 0.43 | 17.6 kDa class II heat shock protein | Protein destination and storage |
| Ta.21335.2.A1_x_at | 0.41 | Heat shock protein 70 family protein | Protein destination and storage |
| Ta.6123.2.S1_at | 0.38 | Heat-shock protein 70T-2 | Protein destination and storage |
| Ta.6964.1.S1_at | 0.48 | Heat shock protein 60-2 | Protein destination and storage |
| TaAffx.18332.1.S1_at | 0.44 | Heat-shock protein 70T-2 | Protein destination and storage |
| TaAffx.107458.1.S1_s_at | 0.47 | Heat shock protein 70 family protein | Protein destination and storage |
| Ta.9140.1.A1_at | 0.49 | Heat shock protein 90.1 | Protein destination and storage |
| Ta.24121.1.S1_x_at | 0.43 | Calcium-binding EF-hand family protein | Protein destination and storage |
| Ta.28735.1.S1_a_at | 0.49 | Chaperone protein htpG family protein | Protein destination and storage |
| Ta.3507.1.S1_at | 0.45 | Serine carboxypeptidase-like 16 | Protein destination and storage |
| TaAffx.87145.1.S1_at | 0.36 | DNAJ heat shock N-terminal domain-containing protein | Protein destination and storage |
| Ta.639.1.S1_at | 0.42 | FKBP-type peptidyl-prolyl cis-trans isomerase family protein | Protein destination and storage |
| Ta.21327.1.S1_x_at | 0.41 | Preprotein translocase Sec, Sec61-beta subunit protein | Transporters |
| Ta.28798.1.S1_x_at | 0.48 | Preprotein translocase Sec, Sec61-beta subunit protein | Transporters |
| Ta.1997.1.S1_at | 0.48 | SecY protein transport family protein | Transporters |
| Ta.1997.2.S1_a_at | 0.48 | SecY protein transport family protein | Transporters |
| Ta.22789.1.S1_at | 0.43 | Vesicle transport V-snare 13 | Transporters |
| Ta.22789.2.A1_at | 0.37 | Vesicle transport V-snare 13 | Transporters |
| Ta.27951.1.A1_at | 0.43 | Major facilitator superfamily protein | Transporters |
| Ta.27951.1.A1_x_at | 0.40 | Major facilitator superfamily protein | Transporters |
| Ta.3938.2.S1_at | 0.43 | Major facilitator superfamily protein | Transporters |
| Ta.5766.1.S1_at | 0.42 | Major facilitator superfamily protein | Transporters |
| Ta.252.1.S1_x_at | 0.46 | Nitrate transporter 2.3 | Transporters |
| Ta.30913.2.A1_at | 0.46 | Phosphate transporter 3;2 | Transporters |
| Ta.30769.1.S1_at | 0.31 | Heavy metal transport/detoxification superfamily protein | Transporters |
| Ta.18672.1.S1_x_at | 0.49 | Metallothionein 2A | Transporters |
| Ta.16450.1.S1_x_at | 0.38 | Delta tonoplast integral protein | Transporters |
| Ta.16450.2.S1_at | 0.41 | Tonoplast intrinsic protein 4;1 | Transporters |
| Ta.9507.2.S1_x_at | 0.47 | Jasmonate-zim-domain protein 1 | Signal transduction |
| Ta.7703.1.S1_a_at | 0.36 | Allene oxide cyclase 1 | Signal transduction |
| Ta.7703.1.S1_x_at | 0.31 | Allene oxide cyclase 4 | Signal transduction |
| Ta.7703.2.S1_x_at | 0.40 | Allene oxide cyclase 1 | Signal transduction |
| Ta.7703.3.S1_x_at | 0.39 | Allene oxide cyclase 1 | Signal transduction |
| Ta.12225.1.S1_x_at | 0.49 | Multiprotein bridging factor 1C | Signal transduction |
| Ta.12225.3.S1_a_at | 0.49 | Multiprotein bridging factor 1C | Signal transduction |
| Ta.22602.1.S1_a_at | 0.26 | Alpha dioxygenase | Signal transduction |
| TaAffx.254.1.S1_at | 0.35 | Pirin | Cell growth |
| Ta.8375.1.S1_at | 0.42 | Cyclin-dependent kinase CDC2C | Cell growth |
| TaAffx.5719.1.S1_at | 0.36 | Methyl esterase 9 | Cell growth |
| Ta.21342.1.S1_x_at | 0.34 | Basic chitinase | Second metabolism |
| Ta.30501.1.S1_at | 0.31 | Basic chitinase | Second metabolism |
| Ta.9580.1.S1_at | 0.45 | Uridine-ribohydrolase 1 | Second metabolism |
| TaAffx.53419.1.S1_s_at | 0.44 | Uridine-ribohydrolase 1 | Second metabolism |
| Ta.8674.1.A1_at | 0.39 | Lanosterol synthase 1 | Second metabolism |
| Ta.12568.2.S1_a_at | 0.49 | Pinoresinol reductase 1 | Second metabolism |
| Ta.24934.3.S1_at | 0.16 | 2-oxoglutarate (2OG) and Fe(II)-dependent oxygenase superfamily protein | Second metabolism |
| Ta.25763.1.S1_at | 0.27 | 2-oxoglutarate (2OG) and Fe(II)-dependent oxygenase superfamily protein | Second metabolism |
| Ta.28233.1.S1_at | 0.38 | 2-oxoglutarate (2OG) and Fe(II)-dependent oxygenase superfamily protein | Second metabolism |
| Ta.3976.1.S1_at | 0.43 | 2-oxoglutarate (2OG) and Fe(II)-dependent oxygenase superfamily protein | Second metabolism |
| Ta.3976.2.S1_x_at | 0.46 | 2-oxoglutarate (2OG) and Fe(II)-dependent oxygenase superfamily protein | Second metabolism |
| Ta.3813.1.A1_at | 0.48 | Cytochrome P450, family 76, subfamily C, polypeptide 2 | Second metabolism |
| Ta.22602.2.S1_x_at | 0.49 | Peroxidase superfamily protein | Defense |
| Ta.29531.2.S1_x_at | 0.46 | Peroxidase superfamily protein | Defense |
| TaAffx.9160.1.S1_at | 0.37 | Peroxidase superfamily protein | Defense |
| TaAffx.39568.2.S1_at | 0.27 | Peroxidase superfamily protein | Defense |
| Ta.14483.1.S1_x_at | 0.36 | Glutathione S-transferase family protein | Defense |
| TaAffx.64766.1.S1_at | 0.39 | Glutathione S-transferase family protein | Defense |
| Ta.8571.1.S1_a_at | 0.47 | Lactoylglutathione lyase / glyoxalase I family protein | Defense |
| Ta.8571.1.S1_x_at | 0.40 | Lactoylglutathione lyase / glyoxalase I family protein | Defense |
| Ta.8571.3.S1_x_at | 0.49 | Lactoylglutathione lyase / glyoxalase I family protein | Defense |
| Ta.26286.1.S1_a_at | 0.46 | Bifunctional nuclease i | Defense |
| Ta.26286.1.S1_x_at | 0.36 | Bifunctional nuclease i | Defense |
| Ta.2107.3.S1_at | 0.45 | Aldehyde dehydrogenase 7B4 | Defense |
| Ta.10996.1.S1_at | 0.47 | Adenine nucleotide alpha hydrolases-like superfamily protein | Defense |
| TaAffx.128595.1.S1_at | 0.28 | Pathogenesis-related 4 | Defense |
| Ta.30739.2.S1_at | 0.27 | Pathogenesis-related protein-like | Defense |
| Ta.233.1.S1_at | 0.37 | Alternative oxidase 1A | Defense |
| TaAffx.15880.1.S1_at | 0.41 | Germin-like protein 4 | Defense |
| Ta.30631.1.S1_at | 0.45 | Ribonuclease 1 | Defense |
| Ta.25026.1.S1_at | 0.21 | Late embryogenesis abundant protein | Defense |
| Ta.449.1.S1_at | 0.28 | Late embryogenesis abundant protein | Defense |
| TaAffx.6564.1.S1_at | 0.35 | Cytochrome P450, family 71, subfamily B | Defense |
| TaAffx.98093.1.S1_at | 0.41 | Cytochrome P450, family 71, subfamily B | Defense |
| Ta.28346.2.S1_x_at | 0.26 | At2g38901/At2g38901 | Unclear |
| Ta.7616.1.S1_s_at | 0.46 | SNARE-like superfamily protein | Unclear |
| Ta.9669.1.S1_at | 0.49 | Phosphate-responsive 1 family protein | Unclear |
| Ta.14491.1.S1_at | 0.27 | Haloacid dehalogenase-like hydrolase (HAD) superfamily protein | Unclear |
| Ta.541.1.S1_at | 0.46 | Cystatin B | Unclear |
| Ta.5557.1.S1_x_at | 0.08 | RmlC-like cupins superfamily protein | Unclear |
| Ta.5624.1.S1_at | 0.50 | Tudor/PWWP/MBT domain-containing protein | Unclear |
| Ta.9997.1.S1_x_at | 0.22 | B12D protein | Unclear |
| Ta.1509.1.S1_s_at | 0.35 | Calcium-dependent phosphotriesterase superfamily protein | Unclear |
| Ta.5444.1.A1_at | 0.32 | Malectin/receptor-like protein kinase family protein | Unclear |
| Ta.8733.2.S1_a_at | 0.46 | Nudix hydrolase homolog 8 | Unclear |
| Ta.8733.2.S1_x_at | 0.44 | Nudix hydrolase homolog 8 | Unclear |
| Ta.22563.1.S1_at | 0.48 | NAD(P)-binding Rossmann-fold superfamily protein | Unclear |
| Ta.22563.1.S1_x_at | 0.48 | NAD(P)-binding Rossmann-fold superfamily protein | Unclear |
| TaAffx.120891.1.S1_at | 0.38 | NAD(P)-binding Rossmann-fold superfamily protein | Unclear |
| Ta.3448.3.A1_at | 0.49 | NAD(P)-binding Rossmann-fold superfamily protein | Unclear |
| Ta.9424.2.S1_at | 0.49 | Fes1A | Unclear |
