# Supplementary material for: Heat Priming Induces Trans-generational Tolerance to High Temperature Stress in Wheat
Source: Front Plant Sci. 2016 Apr 14;7:501. doi: 10.3389/fpls.2016.00501 (PMC4830833; doi:10.3389/fpls.2016.00501)
Supplement: Supplementary file 3 [file Data_Sheet_3.DOCX]

Table S2 Genes differently expressed between the progenies of primed plants (PC) and non-primed plants (NC) under non high-temperature stress during grain filling

| **ProbeSetID** | **Ratio** | **GeneDescription** | **Functional** |
| --- | --- | --- | --- |
| Ta.1385.1.S1_a_at | 2.0 | Methionine synthase 2 | Metabolism |
| Ta.1385.1.S1_x_at | 2.3 | Methionine synthase 2 | Metabolism |
| Ta.1385.2.A1_x_at | 2.0 | Methionine synthase 2 | Metabolism |
| TaAffx.113645.1.S1_at | 2.1 | PLC-like phosphodiesterases superfamily protein | Metabolism |
| TaAffx.82957.1.S1_at | 2.5 | ACT domain repeat 6 | Metabolism |
| Ta.1229.2.S1_at | 2.1 | Threonine aldolase 2 | Metabolism |
| Ta.24768.1.A1_x_at | 2.4 | Nitrite reductase 1 | Metabolism |
| Ta.26144.1.A1_s_at | 2.7 | Fatty acid reductase 1 | Metabolism |
| Ta.22452.1.S1_at | 2.1 | Azelaic acid induced 1 | Metabolism |
| Ta.20784.1.S1_at | 2.5 | Phosphoenolpyruvate carboxylase 2 | Energy |
| Ta.810.2.S1_a_at | 2.2 | Galactose mutarotase-like superfamily protein | Energy |
| TaAffx.53977.1.S1_at | 3.2 | UDP-glucosyl transferase 85A5 | Energy |
| Ta.1028.1.S1_x_at | 2.3 | Hydroxyproline-rich glycoprotein family protein | Cell structure |
| Ta.16171.1.S1_at | 3.1 | Hydroxyproline-rich glycoprotein family protein | Cell structure |
| Ta.18832.1.S1_at | 2.0 | Hydroxyproline-rich glycoprotein family protein | Cell structure |
| Ta.19544.1.S1_at | 2.1 | Hydroxyproline-rich glycoprotein family protein | Cell structure |
| Ta.19544.1.S1_x_at | 2.0 | Hydroxyproline-rich glycoprotein family protein | Cell structure |
| Ta.13337.2.S1_at | 2.1 | Xyloglucan endotransglucosylase/hydrolase 12 | Cell structure |
| Ta.13337.2.S1_x_at | 2.3 | Xyloglucan endotransglucosylase/hydrolase 12 | Cell structure |
| TaAffx.78449.1.S1_at | 2.1 | Wall-associated kinase family protein | Cell structure |
| Ta.3746.1.S1_at | 2.4 | Actin binding | Cell growth |
| Ta.7046.1.S1_at | 2.2 | NAC domain containing protein 36 | Cell growth |
| Ta.3395.1.S1_at | 2.3 | K-box region and MADS-box transcription factor family protein | Transcription |
| Ta.23763.1.S1_at | 3.2 | PLAT/LH2 domain-containing lipoxygenase family protein | Transcription |
| TaAffx.64645.1.A1_s_at | 2.0 | Transducin family protein / WD-40 repeat family protein | Transcription |
| TaAffx.9432.1.A1_at | 2.2 | Eukaryotic elongation factor 5A-3 | Protein sythesis |
| TaAffx.56014.2.S1_x_at | 2.1 | Eukaryotic aspartyl protease family protein | Protein destination and storage |
| TaAffx.81921.1.S1_at | 2.5 | Cysteine proteinases superfamily protein | Protein destination and storage |
| TaAffx.89907.1.S1_x_at | 2.2 | Protein-l-isoaspartate methyltransferase 2 | Protein destination and storage |
| Ta.4117.3.S1_x_at | 2.1 | Glycosyltransferase family 61 protein | Transporters |
| TaAffx.25652.1.S1_at | 2.0 | Amino acid permease 3 | Transporters |
| Ta.5324.1.A1_at | 2.4 | ZRT/IRT-like protein 2 | Transporters |
| TaAffx.107485.1.S1_at | 2.1 | Nucleotide-diphospho-sugar transferases superfamily protein | Transporters |
| Ta.30769.1.S1_at | 2.1 | Heavy metal transport/detoxification superfamily protein | Transporters |
| TaAffx.7917.1.S1_at | 2.0 | Undecaprenyl pyrophosphate synthetase family protein | Transporters |
| TaAffx.2601.1.S1_at | 2.7 | Organic cation/carnitine transporter 3 | Transporters |
| Ta.12893.1.S1_at | 2.5 | Hemoglobin 1 | Transporters |
| TaAffx.28241.1.S1_at | 2.3 | Protein kinase superfamily protein | Signal transcription |
| TaAffx.39446.1.S1_at | 4.1 | Histidine kinase | Signal transcription |
| TaAffx.86449.1.S1_at | 7.4 | S-locus lectin protein kinase family protein | Signal transcription |
| TaAffx.59867.1.S1_at | 34.3 | Ethylene-forming enzyme | Signal transcription |
| Ta.26230.1.S1_at | 2.1 | Peroxidase superfamily protein | Defense |
| Ta.26230.1.S1_x_at | 2.2 | Peroxidase superfamily protein | Defense |
| Ta.26230.2.S1_at | 2.0 | Peroxidase superfamily protein | Defense |
| TaAffx.110012.1.S1_at | 2.1 | DCD (Development and Cell Death) domain protein | Defense |
| TaAffx.31943.1.S1_at | 3.0 | Pathogenesis-related protein-like | Defense |
| Ta.21438.1.A1_at | 2.2 | Cytochrome P450, family 71, subfamily B, polypeptide 35 | Defense |
| Ta.22556.1.S1_x_at | 2.3 | Disease resistance-responsive (dirigent-like protein) family protein | Defense |
| Ta.1643.1.S1_at | 2.1 | Laccase 12 | Second metabolism |
| Ta.21342.1.S1_x_at | 2.1 | Basic chitinase | Second metabolism |
| Ta.3976.2.S1_x_at | 2.8 | 2-oxoglutarate (2OG) and Fe(II)-dependent oxygenase superfamily protein | Second metabolism |
| Ta.24934.3.S1_at | 2.2 | 2-oxoglutarate (2OG) and Fe(II)-dependent oxygenase superfamily protein | Second metabolism |
| Ta.16497.1.S1_at | 2.4 | Urophorphyrin methylase 1 | Second metabolism |
| TaAffx.59384.2.S1_s_at | 2.2 | RNI-like superfamily protein | Unclear |
| TaAffx.78951.1.S1_at | 2.6 | Putative uncharacterized protein AT4g09360 | Unclear |
| Ta.277.1.S1_at | 0.20 | Limit dextrinase | Metabolism |
| Ta.8866.1.S1_at | 0.49 | lipid-transfer protein | Metabolism |
| TaAffx.119272.1.S1_at | 0.43 | Homocysteine S-methyltransferase family protein | Metabolism |
| TaAffx.57100.1.S1_at | 0.24 | Glycerol-3-phosphate acyltransferase 5 | Metabolism |
| Ta.7740.1.A1_at | 0.42 | Alpha/beta-Hydrolases superfamily protein | Metabolism |
| Ta.30702.1.S1_x_at | 0.48 | Light-harvesting chlorophyll-protein complex II subunit B1 | Energy |
| Ta.8584.1.S1_at | 0.43 | Beta-1,3-glucanase 3 | Energy |
| Ta.9096.2.S1_x_at | 0.49 | Phosphoenolpyruvate carboxylase 1 | Energy |
| TaAffx.131312.1.S1_at | 0.37 | Alpha carbonic anhydrase 7 | Energy |
| Ta.9530.1.S1_at | 0.33 | Glucose-6-phosphate/phosphate translocator 2 | Energy |
| Ta.30327.2.A1_at | 0.30 | UDP-glucosyl transferase 88A1 | Energy |
| Ta.8942.1.S1_at | 0.28 | Hydroxyproline-rich glycoprotein family protein | Cell structure |
| Ta.667.2.S1_a_at | 0.31 | Integral membrane HPP family protein | Transcription |
| Ta.28659.1.S1_x_at | 0.29 | Serine protease inhibitor, potato inhibitor I-type family protein | Protein destination and storage |
| TaAffx.132498.1.S1_at | 0.46 | Serine protease inhibitor, potato inhibitor I-type family protein | Protein destination and storage |
| TaAffx.30389.1.S1_at | 0.34 | Mitochondrial HSO70 2 | Protein destination and storage |
| Ta.8856.1.A1_at | 0.45 | Plant calmodulin-binding protein-related | Signal transduction |
| TaAffx.21593.1.S1_at | 0.47 | Pyridoxal phosphate (PLP)-dependent transferases superfamily protein | Transporters |
| TaAffx.82806.1.S1_at | 0.37 | ABC transporter G family member 40 | Transporters |
| Ta.4821.1.A1_at | 0.37 | ABC transporter G family member 40 | Transporters |
| Ta.28233.1.S1_at | 0.40 | 2-oxoglutarate (2OG) and Fe(II)-dependent oxygenase superfamily protein | Second metabolism |
| Ta.3813.1.A1_at | 0.40 | Cytochrome P450, family 76, subfamily C, polypeptide 2 | Second metabolism |
| TaAffx.108556.1.S1_x_at | 0.50 | Pathogenesis-related 4 | Defense |
| TaAffx.108908.1.S1_x_at | 0.34 | Osmotin 34 | Defense |
| TaAffx.19033.1.S1_at | 0.40 | Tetratricopeptide repeat (TPR)-like superfamily protein | Unclear |
| Ta.5978.2.A1_at | 0.42 | CBS domain-containing protein with a domain of unknown function | Unclear |
| Ta.5666.1.S1_at | 0.18 | Hipl2 protein precursor | Unclear |
| Ta.29578.1.S1_s_at | 0.37 | Hemoglobin 1 | Unclear |
